# Novel recombinant Mce-truncated protein based ELISA for the diagnosis of *Mycobacterium avium* subsp. *paratuberculosis* infection in domestic livestock

**DOI:** 10.1371/journal.pone.0233695

**Published:** 2020-06-01

**Authors:** Zahra Hemati, Masoud Haghkhah, Abdollah Derakhshandeh, Kundan Kumar Chaubey, Shoor Vir Singh

**Affiliations:** 1 Department of Pathobiology, School of Veterinary Medicine, Shiraz University, Shiraz, Iran; 2 Animal Health Division, Central Institute for Research on Goats, Makhdoom, Farah, Mathura Uttar Pradesh, India; Jamia Hamdard, INDIA

## Abstract

Johne’s disease (JD) is an infectious wasting condition of ruminants caused by *Mycobacterium avium* subsp. *paratuberculosis* (MAP) in domestic livestock of every country that has been investigated. Controlling JD is problematic due to the lack of sensitive, specific, efficient, and cost-effective diagnostic tests. A major challenge in the development of diagnostics like ELISA is the selection of an ideal antigen/(s) that is pathogen-specific and allows sensitive recognition. Therefore, the purpose of this study was to identify and use Mce-truncated protein-based ELISA assay for the diagnosis of MAP infection with high sensitivity and specificity. *In silico* epitope prediction by epitope mapping throughout the whole length of MAP2191 protein revealed that C-terminal portion of this protein presented potential T- and B-cell epitopes. Therefore, a novel Mce-truncated protein encoded by the selected region of *MAP2191* gene was expressed, purified with Ni-NTA gel matrix and confirmed by SDS PAGE and western blot. A profiling ELISA assay was developed to evaluate sera from MAP infected and non-infected ruminant species for antibodies against Mce-truncated protein to infer the immunogenicity of this protein in the host. Using this Mce protein-based ELISA, 251 goats, 53 sheep, 117 buffaloes, and 33 cattle serum samples were screened and 49.4, 51.0, 69.2, and 54.6% animals, respectively, were found positive. Comparing with i-ELISA, the new Mce-based ELISA kit showed a relatively higher specificity but suffered from slightly reduced sensitivity. Mce-based ELISA excluded apparently false positive results of i-ELISA. Mce protein was found to be antigenic and Mce-ELISA test could be employed as a diagnostic test for JD in domestic livestock in view of the a relatively higher specificity and accuracy. The antigenic potential of Mce antigen can also be exploited for the development of a new vaccine for the control of MAP infection.

## Introduction

*Mycobacterium avium* subsp. *paratuberculosis* (MAP) is the cause of chronic wasting disease in domestic livestock species commonly known as paratuberculosis or Johne’s disease (JD) [1, 2]. JD has the widest host range and has been isolated from domestic livestock, wild ruminants, and other animal species, including primates and human beings [3, 4–6]. The disease inflicts major economic losses to livestock production system and dairy industry by lowering productivity, both in terms of quality and quantity, of milk, meat, fiber and skin, by way of increased morbidity, mortality and early cullings [1, 6]. Large number of studies reported high bio-load of MAP in domestic livestock and in their milk [7, 8] and other secretions. MAP has also been associated with a number of incurable human illnesses such as Crohn’s disease / Inflammatory Bowel Disease, diabetes, rheumatoid arnthritis, thyroids, and other auto-immune type disorders [1, 2, 9]. Control and eradication of JD are challenging due to the insidious nature, long incubation period, and lack of rapid and sensitive diagnostic tests [10]. With the scale and complexity of the expanding problem of MAP, pressure is increasing to find a way to control MAP infections in animals in order to rescue high milk yielding breeds of domestic livestock, and reduce production and economic losses in the livestock, dairy industry and animal farming, and impending threat for large scale human infection by consumpton of milk and milk products contaminated with MAP bacilli [6, 11, 12].

Recent immuno-informatics tools have made *in silico* analysis possible; such analysis has been used successfully to predict the immune epitopes in the pathogens and identify immunogenic proteins [13]. Presently, ideal major antigen candidates for MAP for efficient immuno-diagnosis and immunization are not available [11, 13]. There is need for the identification of immunogenic candidate antigens, which can be assessed for the development of efficient diagnostic tests and also as effective vaccine candidates against MAP infection [11]. Mammalian cell entry (Mce) proteins are among the virulence-related proteins which are functionally analogous to ABC transporters and are thought to function in lipid uptake system [14–18]. It was shown that Mce proteins have possible role in virulence of *Mycobacterium tuberculosis* (MTB) [15, 19–21]. Information is limited to the individual Mce protein expression in terms of their contribution to virulence in other Mycobacteria such as MAP. In MAP K-10 reference genome, there are eight separate *mce* genes [14, 22, 23]. *Mce*5 operon of MAP possesses a cluster of six homologs of the *mce*-family (*MAP2189* to *MAP2194*) genes predicted to encode proteins with potentially useful metabolic functions [24–26]. *Mce*5 cluster has previously been identified as an important player for invasion and survival within macrophages, and possibly for the virulence of MAP [25]. Expression studies revealed that the purified Mce family of proteins has the ability to elicit antibody responses during *Mycobacterium tuberculosis* infection in human [27, 28].

Present study identified immuno-dominant candidate antigens using immune-informatics analysis (T- and B-cell epitopes analysis) for the development of improved diagnostic tests. Mce-truncated protein is a partial part of Mce protein (MAP2191) which has been proposed to be expressed from the truncated gene of *MAP2191*, and used as the coating antigen in the ELISA platform for the sero-diagnosis of JD using animal sera from naturally infected and infection free controls.

## Materials and methods

### Ethics statement

Central Institute for Research on Goats (CIRG), Makhdoom, Mathura ethical committee chaired by Member Secretary, Institutional Animal Ethics Committee (IAEC) under the supervision of central Committee for the Purpose of Control and Supervision of Experiments on Animals (CPCSEA), New Delhi has approved the experiments which were performed under the project [grant number 5/8/5/28/TF/2013/ECD-I], awarded by Indian Council of Medical Research (ICMR), New Delhi, India. IAEC vide reference number IAEC/CIRG/16-17 dated: 12.05.2016, confirmed that this project do not have any ethical issue. Samples were collected only for laboratory analyses and avoided unnecessary pain and suffering to animals and were not from any endangered or protected species.

### Serum samples

Serum samples were collected from four domestic livestock species (251 goats, 53 sheep, 117 buffaloes, and 33 cattle) suspected for MAP infection. Animals sampled were both from farm and farmer’s herds and flocks located in two states {(Uttar Pradesh (UP) and Madhya Pradesh (MP)} of country. A total of 251 goats (40-IGFRI- Indian Grassland and Fodder Research Institute, Jhansi, UP; 91-SADIL- State Anima Disease Investigation Laboratory, Bhopal, MP; 120-Veterinary college, Mhow, MP), 53 sheep (53-IGFRI- Indian Grassland and Fodder Research Institute, Jhansi, UP), 117 buffaloes (117-Veterinary college, Mhow, MP), and 33 cattle (33- SADIL- State Anima Disease Investigation Laboratory, Bhopal, MP) were included in this study. Animals were in variable age mainly from females and they have mixed physical conditions and 30–35% of the animals were suffering from clinical to advance clinical JD. All these animals were ear-tagged for identification.For the collection of blood samples goats and sheep by restraining manually by side of chest and gently holding the the head towards one side. Cattle and buffaloes were properly restrained in cattle crush and blood (2–3 ml) was collected in vacutainers, after properly sterilizing the jugular vein with 70.0% ethyl alcohol. Institute Animal Ethics committee (IAEC) of the CIRG, Mathura, UP, India has approved the technical program of the project. Samples were stored at -20°C until screened. Status of the animals with respect to MAP infection was determined using ‘Gold Standard’fecal culture, fecal microscopy and fecal IS*900*-PCR before screening through i-ELISA and Mce-ELISA.

### Morphological and molecular characterization

Reference strain of MAP ‘S 5’ Indian Bison Type was provided by CIRG, Mathura, UP, India. Strain used in this study was recovered from an advance case of JD (terminally sick, extremely weak and recumbent) in a Jamunapari breed of goat [29], which later succumbed to disease [30]. The MAP strain was sub-cultured and colonies identified morphologically on Herrold’s egg yolk (HEY) agar medium, mycobactin dependency and acid fast (Ziehl-Neelsen) staining of smears. Method for fecal culture and decontamination methods explained in the text. Briefly, ingredients of HEYM weighed (Mineral salt solution—MSS) and pH was made 7.8 after adding glycerol. Agar powder added and MSS (870 mil) was autoclaved. Egg yolk (120 ml from 8 eggs) harvested after sterilizing in isopropanol. Antibiotic free eggs from local poultry farmers used. Harvested yolk and 5 ml of 2.0% malachite green were added to MSS at 560C and HEYM was stirred for 2 hr at 500C. Mycobactin J was added to HEYM and dispensed. Fecal smears were made from middle layer after triturating feces (2 gm) in 10 ml sterilized water and centrifuged at 5000 rpm for 60 min. Molecular characterization of the strain was done using IS*900*, IS*1311* PCR and IS*1311* PCR-REA [29].

### Identification of antigenicity *mce* gene by in-silico analysis

*In Silico* analysis of the MAP2191 protein was carried out using CLC Genomics Workbench 7.5.1 program (CLC bio, QIAGEN, Germany) and Major histocompatibility complex (MHC) binding peptide prediction algorithms through Immune Epitope Database and Analysis Resource (IEDB-AR) site (http://www.iedb.org/). MAP2191 protein full-length coding sequence was subjected to a basic local alignment search (BLASTp) analysis at the National Center for Biotechnology Information (NCBI) GenBank. After that T cell MHC class I and II epitopes prediction were performed using the IEDB-AR analysis consensus tool, which combines predictions from the Artificial neural network (ANN), Stabilized matrix method (SMM) and combLib [13, 31–34]. T cell epitopes prediction were calculated on the basis of their binding affinity to MHC alleles of the mouse, using the half-maximal inhibitory concentration (IC50) values as follows: IC50s <50 nm (high-affinity binding); IC50s <500 nm (intermediate-affinity binding), and IC50s <5,000 nm (low-affinity binding). A lower IC50 for MAP2191 protein indicates a higher affinity of binding to host MHC alleles. Mouse MHC alleles H-2Db, H-2Dd, H-2Kb, H-2Kd, H-2Kk, and H-2Ld were used for peptide binding affinity to MHC-I epitopes and H-2IAb, H-2IAd, and H-2IEd were used for peptide binding affinity to MHC-II epitopes [31–33]. For the prediction of linear and conformational B cell epitopes, the MAP2191 protein sequence was subjected to an ElliPro suite [35]. We obtained a score for each predicted epitope defined by PI average over each amino acid residue present in the MAP2191 protein. Predicted B cell epitopes of MAP2191 protein had scores of between 0.5 and 0.9; the minimum prediction cutoff score was set at 0.80.

### Amplification and cloning of *mce*-truncated gene in TA-cloning vector

*Mce*-truncated gene (Accession number: MG754208) was successfully amplified with the help of primers designed to have *Bam*HI and *Hind*III restriction enzyme sites (Table 1). Thermocycling was done in Veriti 96 well thermal cycler (Applied Biosystem), with initial hot start denaturation step for 5 min at 94°C, followed by 37 cycles of 94°C for 1 min, 58°C for 30 sec, and 72°C for 1 min. Subsequently, a final extension at 72°C for 10 min was carried out. Purified *mce-*truncated was confirmed by nucleic acid sequencing and cloned with pTZ57R/T cloning vector. Ligation products were successfully transformed into *E*. *coli* DH5α/*E*. *coli* XL10-Gold ultra-competent cells followed by plating on Luria Bertani (LB) agar (Merck, Germany) containing X-gal, IPTG, Ampicillin, Tetracycline, and Chloramphenicol. Final confirmation of purified clones was done using restriction digestion followed by sequencing.

**Table 1:** Primers used for characterization of *Mycobacterium avium* subsp. *paratuberculosis*.

Target	Primer	Primer sequence	Product size (bp)
MAP2191/ truncated	Forward	5’ACGGGATTCACCCTCAATCAATCGCTGG 3’-*Bam*H1	621 (MAP Mce)
	Reverse	5’CTAAAGCTTTCATCGCGAACCGCCCGGGATG 3’- *Hind*III	

### Cloning and confirmation of *mce*-truncated gene in expression vector

Expression plasmid was generated by cloning *mce*-truncated gene of MAP using pET28a (+) expression vector as per Chaubey et al. (2018) [36] with some modifications. Prior to ligation reaction, pET28a vector was restricted with *Hind*III and *Bam*HI endonuclease, de-phosphorylated by a fast alkaline phosphatase enzyme (Fermentas, USA) with 10X FastAP buffer, and gel purified. For ligation reaction, *mce*-truncated gene fragment was digested from a pTZ57R/T vector with *Hind*III and *Bam*HI, gel purified, and ligated with de-phosphorylated and digested using pET28a plasmid. Ligation product was transformed into *E*. *coli* DH5α/XL-10 competent cells followed by plating on LB-agar containing 50 μg ml^-1^ Kanamycin. Six to ten colonies were picked for *mce-*truncated colony PCR for the confirmation of the insert. Plasmid DNA of concern clones was isolated and digested with *Hind*III and *Bam*HI enzymes. In order to further confirm the insert, one of the confirmed pET28a-*mce*-truncated cloning vectors was sequenced using T7 universal primers.

### Expression and purification of Mce-recombinant protein

To express Mce-truncated protein, cloned vector was further transformed into *E*. *coli* Rosetta and *E*. *coli* BL-21 (DE3) competent cells; a single colony was picked up and propagated in 10 ml fresh Luria Bertani broth supplemented with Kanamycin (50 μg ml^-1^) at 37°C with shaking (180 rpm) overnight. For expression, 500 ml LB-broth containing Kanamycin were inoculated with 1% overnight grown culture and propagated until the absorbance reached approximately 0.5 at 600 nm. In the next step, cells were induced with different concentrations of IPTG and cultures were grown at different temperatures for different time intervals. One ml of uninduced cells (as negative control) were collected just before the addition of IPTG and from the induced cells at different time intervals, i.e. 2, 4, 6, 8, 10, 20 and 24 hrs. post induction. Collected culture was centrifuged and the pellet was harvested.

Purification of His-tagged Mce-truncated protein was carried out by affinity chromatography on a nickel-nitrilo triacetic acid (Ni-NTA) gel matrix (Qiagen, USA) using batch method. In brief, Rosetta and BL21 cells, harboring *mce*-truncated pellets, were suspended by gentle stirring in 15 ml equilibration buffer (50 mm Tris. HCl, 200 mm NaCl, 5mm DTT, 1mm PMSF, pH 8.0). The 14 ml cell suspension was lysed on ice using sonication at 30% amplitude for 20 cycles (20 sec pulse on, 20 sec pulse off). Then, a 50 ml conical tube containing 12 ml crude lysate was equilibrated with 1ml of pre-washed Ni-NTA resin in a horizontal position for 8 hours at 4°C while mixing. After incubation, the equilibrated suspension was centrifuged (2000 rpm, 10 min, 4°C) and the pellet was washed three times with wash buffer. Captured Mce-truncated proteins were eluted by increasing the imidazole concentration to 250 mM in the elution buffer.

### SDS-PAGE and western blotting

Expression of Mce-truncated protein was analyzed by 12% Sodium Dodecyl Sulfate Polyacrylamide Gel Electrophoresis (SDS-PAGE) to investigate the desired size of expressing proteins. For western blotting, Mce-truncated protein band was transferred to nitrocellulose membrane (GE water and process technologies) with a Transblot Turbo® (Biorad, USA) apparatus operated at 60 volts for 2 hrs. The unbound membrane sites were first blocked by incubation for 30 min in blocking buffer (3% skimmed milk powder) at room temperature (RT). Blocking buffer was removed, followed by three rounds of washing with phosphate buffer saline with tween 20 (PBST buffer). The membrane was dipped with anti His Tag monoclonal antibody (Sigma-Aldrich, Germany; 1:10000 dilutions) for 1 hr. at RT. After 3 final washing with PBS- Tween-20, the membrane was immersed in 0.02% substrate solution (diaminobenzidine (DAB) / Roche Applied Science) suspended in PBS containing 0.03 Hydrogen Peroxide. Color was developed by incubation in the dark at RT for 5–10 min until a deep brown band appeared. Concentration of the purified Mce-truncated protein was estimated by Bradford protein estimation assay. In Addition, another Western blotting was also performed on SDS-PAGE gel with sera from tuberculosis infected animals, to determine the cross reactivity of Mce-truncated protein. Serum samples used to check cross reactivity of protein, were obtained from six tuberculin positive cattle.

### Recombinant Mce-truncated protein (antigen)

Recombinant protein Mce (MAP2191) was identified on the basis of it’s moleculara weight by SDS-PAGE and it’s immunoreactivity in western blot assay.

### Mce-ELISA

Mce-ELISA was performed to quantify titer of the antibody in serum samples of naturally infected, suspected, and non-infected (Healthy) animals. Concentrations of serum (1:50), antigen and conjugate (1:8000 for goats and sheep; 1:6000 for cattle and buffaloes) were optimized by checker board analysis. Flat bottom 96-well Microtiter plates (Cat. No. 655061, Greiner bio-one, made in Germany) were coated with 100 μl of recombinant Mce-truncated protein containing 0.02 μg of antigen in coating buffer. Coated plates were incubated over-night at 4°C. After incubation, antigen-coated plates were washed one time with washing buffer (1X PBS containing 0.05% [v/v] Tween-20). Uncoated surfaces were blocked (100 μl/well) with blocking buffer (3.0% skimmed milk) for one hour at 37°C. Following three washes with washing buffer, 100 μl of diluted serum (1:50) in serum dilution buffer (1.0% BSA) were added in duplicate to each well. Plates were incubated at 37°C for one hour, emptied, and washed three times with washing buffer. Secondary antibodies used in this assay were peroxidase-labelled anti-species whole IgG antibody produced in rabbits (Cat. numbers A8919 for goats/sheep and A5295 for cattle/buffaloes, Sigma-Aldrich, Inc.), at 1:8000 dilution for goats and sheep and 1:6000 for cattle and buffaloes in 1xPBS. 100 μl of secondary anti-species antibody was added to each well and incubated for 50 minutes at 37°C. After washing four times, 100 μl of chromogenic substrate solution of O-Phenylenediamine dihydrochloride (OPD) (Cat. number P3804, Sigma-Aldrich, Inc.) was prepared as per manufacturer’s recommendation and added to each well. Plates were incubated for 10–15 minutes in the dark at 37°C. Extent of the color development (optical density) was measured at the absorbance of 450 nm using Bio-RAD *i-*mark ELISA plate reader. Positive and negative controls were selected from the animals that were fully characterized on cultural/ morphological characteristics (Ziehl-Neelsen staining), molecular characteristics (IS*900* PCR, IS*1311*PCR and Real time PCR), serological screening (ELISA and Dot-ELISA) and other tests (LAT and FAT).

### Indigenous ELISA kit (i-ELISA)

A robust and extensive validated indigenous ELISA kit based on semi-purified Protoplasmic antigen (sPPA) prepared from the characterized strain (S 5) 'Indian Bison Type' bio-type of MAP of goat origin (provided by CIRG) was used for the comparative studies. PPA used in this kit was harvested as whole cell sonicate. Lysate was centrifuged and supernatant was used as antigen after protein estimation. Initially, i-ELISA was developed for the screening of goats and sheep [30, 37, 38] and has since been standardized for screening cattle and buffaloes [39]. Serum samples were used in 1:50 dilution and anti-species horseradish peroxidase conjugates (Sigma Aldrich, USA) at the dilution of 1:5000 for goats and sheep and 1:4000 for cattle and buffaloes in 1xPBS. Serum samples from culture positive and negative animals were used as positive and negative controls, respectively.

S/P ratio for Mce: Optical densities (OD) were expressed as sample-to-positive (S/P) ratios as per Collins (2002) by the following formula (Table 2).

$$S/P\;ratio\;value=\frac{\mathrm{OD}\;\mathrm{at}\;450\;\mathrm{nm}\;\mathrm{of}\;\mathrm{test}\;\mathrm{serum}-\mathrm{OD}\;\mathrm{at}\;450\;\mathrm{nm}\;\mathrm{of}\;\mathrm{negative}\;\mathrm{control}}{\mathrm{OD}\;\mathrm{at}\;450\;\mathrm{nm}\;\mathrm{of}\;\mathrm{positive}\;\mathrm{control}-\mathrm{OD}\;\mathrm{at}\;450\;\mathrm{nm}\;\mathrm{of}\;\mathrm{negative}\;\mathrm{control}}$$

Sensitivity and Specificity:

Sensitivity = True Positive X 100 / True Positive + False Negative

Specificity = True Negative X 100 / True Negative + False Positive

**Table 2:** S/P ratios (ELISA OD values and the status Johne’s disease in animals.

S No.	Calculated value of S/P Ratio	Johne’s disease status
1	0.00–0.09	Negative (N)
2	0.10–0.24	Suspected or Borderline (S)
3	0.25–0.39	Low Positive (LP)
4	0.4–0.99	Positive (P)
5	1.0–10.0	Strong Positive (SP)

### Statistical analyses

Statistical differences between the results of Mce-ELISA and i-ELISA were analyzed by McNemar’s and kappa agreement was applied to measure the statistical significance between the results of the two tests (GraphPad software, USA).

## Results

### Morphological and molecular characteristics of MAP

**Fig 1:**
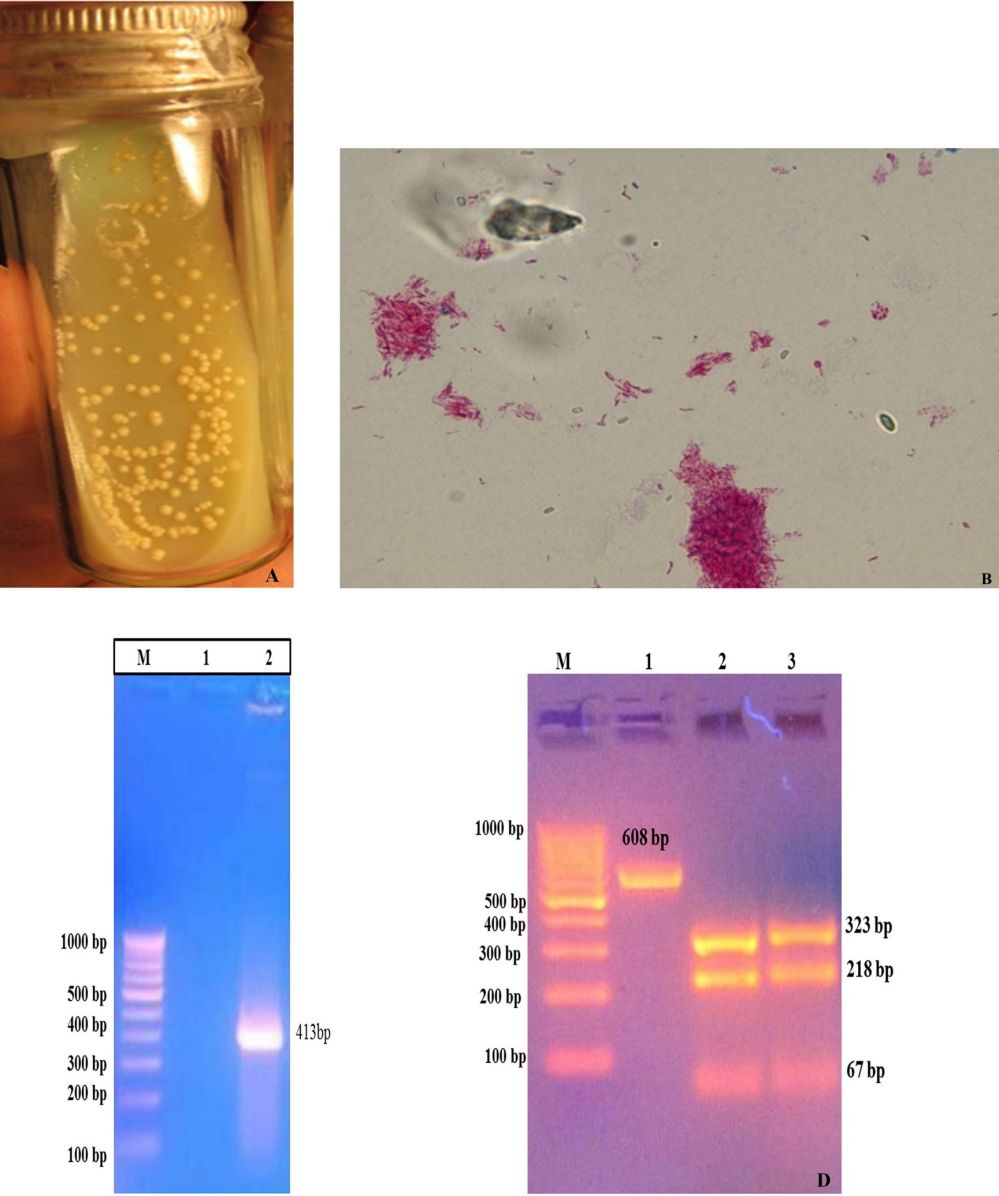
Characterization of sub-cultured of MAP reference strain (S5) on HEY medium. (A) Characterization of sub-cultured MAP ‘S 5’ colonies (B) Microscopic view of acid fast bacilli (indistinguishable to MAP) under oil immersion (100X). Molecular Characterization of MAP ‘S5’ DNA: (C) IS*900* PCR: Lane M- 100 base Marker (#SM0243, Fermentas), Lane 2: IS*900* PCR product; (D) IS*1311* PCR and IS*1311*-REA genotyping: Lane M: 100 base Marker (#SM0243, Fermentas), Lane 1: IS*1311* PCR product, Lane 2: Digested PCR product of IS*1311* from Positive control, Lane 3: Digested PCR product of IS*1311* from sub-cultured colonies.

Colonies of the MAP reference strain (S 5) appeared on the slants of HEY medium. Colonies were small, straw color, translucent, hemispherical, raised with nipple, smooth, and glistening (Fig 1A). Staining of smears from MAP colonies by ZN-stain exhibited short pink staining cocco-bacillary nature (Fig 1B). Yield of specific PCR products in IS*900* PCR, confirmed the presence of MAP (Fig 1C and 1D).

### Bioinformatics analysis

Alignment and comparison of MAP K-10 and MAP (S 5) strain sequences illustrated highly conserved sequences, likely to be cross-reactive, as most epitopes were shared. Moreover, *MAP2189*-*MAP2194* cluster organization was also conserved in both the strains. Based on Bioinformatics analysis, *MAP2191* gene from MAP was of 1065bp and encodes for a typical linear Mce protein that has 354 amino acids with an estimated molecular weight of 37.510 kDa. Possible secondary structure of the MAP2191 protein was predicted using SOPMA website and analyzed results revealed that this protein consisted of 41.53% α-Helix, 16.95% extended strand, 11.58% β-turn and 29.94% random coils. C-terminal portion of MAP2191 protein was successfully expressed and purified in this study. Partial Mce-truncated protein sequence (amino acids 154 to 354) was chosen based on the predicted sequence to contain most dominant B- and T-cell epitopes. CLC software analysis also revealed that selected region of MAP2191 protein had high antigenic index and was more hydrophilic.

### Amplification, cloning, and sub-cloning of *mce*-truncated gene

**Fig 2:**
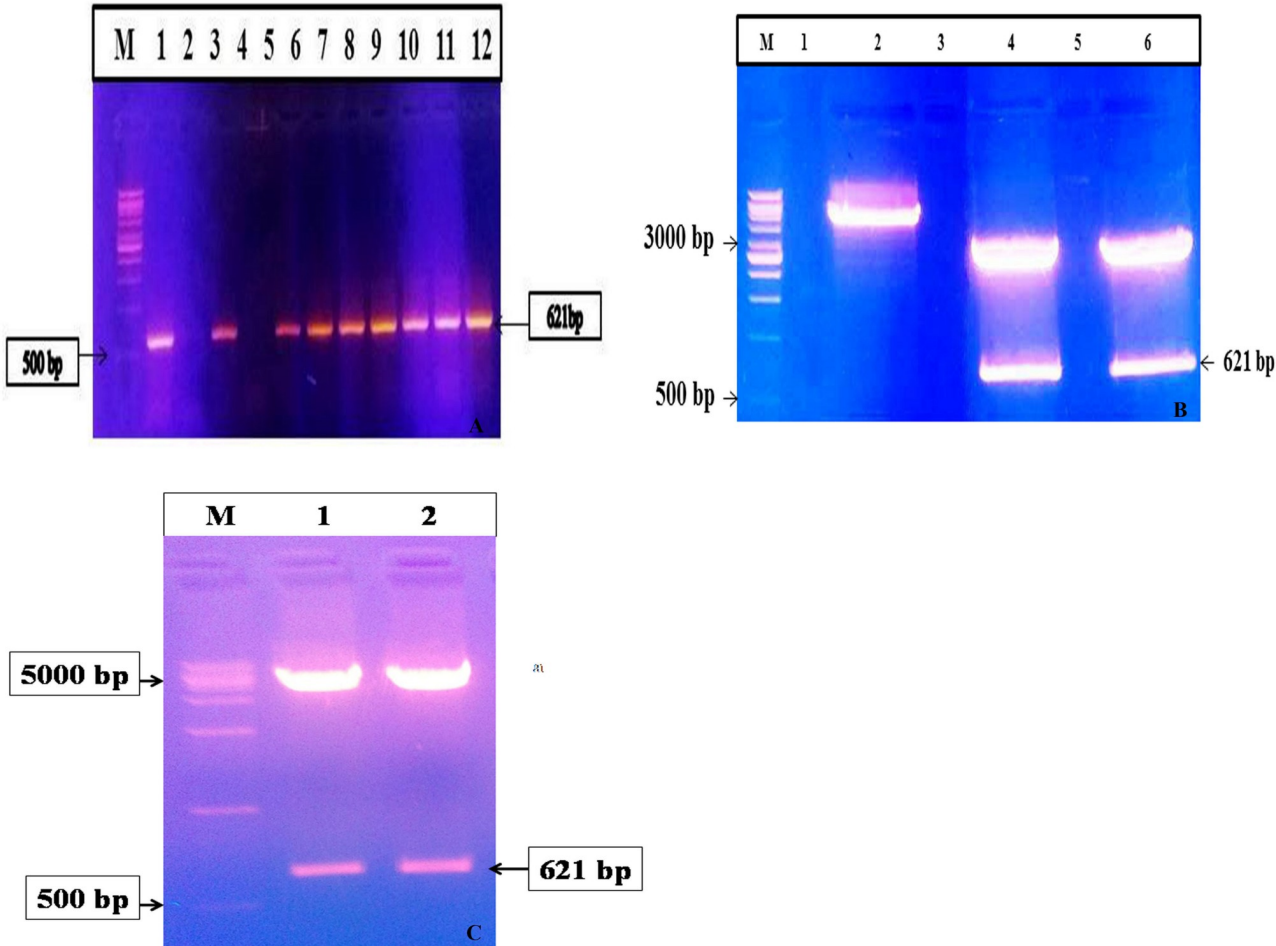
(A) Colony PCR for MAP *mce*-truncated gene, Lane M: 1kb DNA ladder (#SM0313, Fermentas), Lane 1: Positive control (MAP Indian Bison type DNA), Lane 2: Negative control (Nuclease Free Water), Lane 4: Negative in colony PCR for MAP *Mce*-truncated genes, Lanes 3 and 5–12 positive in colony PCR for MAP *Mce*-truncated genes; (B) Restriction digestion of confirmed colony PCR positive PTZ57R/T *mce*-truncated clones: Lane M: 1kb DNA ladder (#SM0313, Fermentas), Lane 2: Double digested pET28a plasmid, Lanes 4 and 6: Positive clones of PTZ57R/T *mce*-truncated plasmid; (C) Restriction digestion of confirmed colony PCR positive pET28a *mce*-truncated clones; Lane M: 1kb DNA ladder (RBC), lanes 1 and 2: Positive clones of PTZ57R/T *mce*-truncated plasmid.

Whole DNA was isolated from MAP (S 5) strain, and target region of *MAP2191* gene was successfully amplified by PCR. Agarose gel electrophoresis of PCR products showed a single band of 621bp amplicon (603bp *mce* gene and extra 18bp were used to restrict sites and bring the coding sequence in-frame) as positive for the truncated gene (Fig 2A). *Mce*-truncated amplicon was sequenced and the sequence results were aligned using BLASTp analysis; no mismatch was reported in sequence data. The amplified *mce*-truncated fragment was purified and ligated into pTZ57R/T TA cloning vector and transformed into *E*. *coli* competent cells. Transformants were confirmed for the presence of *mce*-truncated insert by specific colony PCR and restriction analysis (Fig 2B). Gene sequencing results confirmed the presence of *mce*-truncated gene segment in pTZ57R/T vector. Purified *mce*-truncated gene from TA-cloning vector was sub-cloned into pET28a (+) expression vector between the restriction sites *Bam*HI and *Hind*III and transformed into XL10-Gold ultra-competent cells for the high percentage of recombinant plasmid yields. Presence of the insert was approved by colony PCR and double digestion using restriction enzymes (Fig 2C). Gene sequencing results confirmed the presence of *mce*-truncated gene in the pET28a vector.

### Expression and purification of Mce-recombinant protein

**Fig 3:**
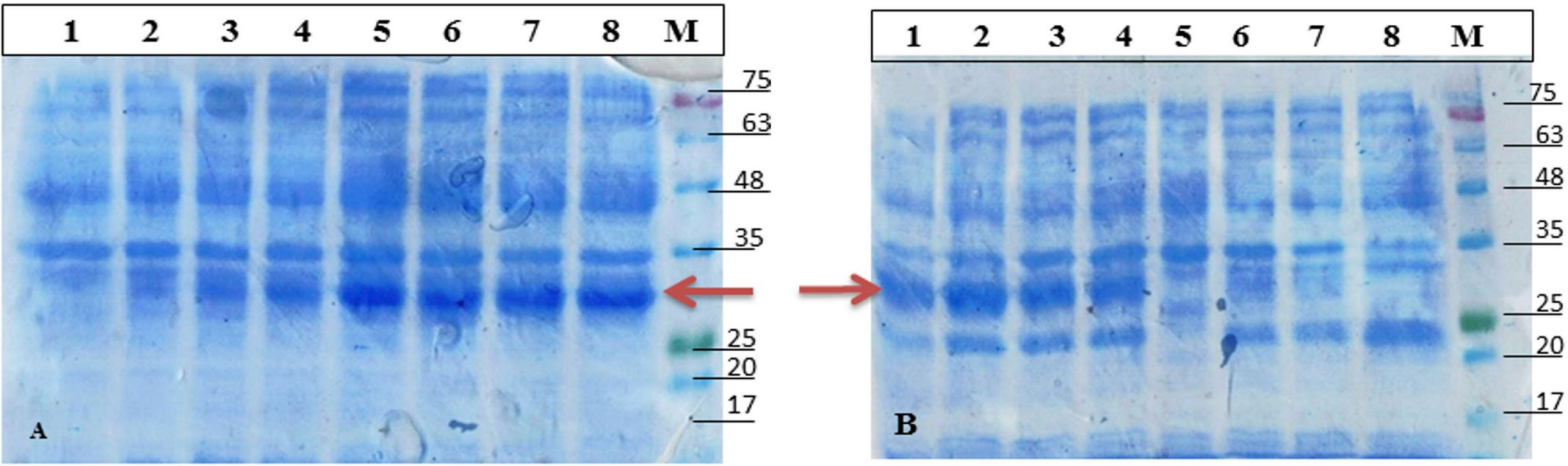
Optimization condition for Mce-truncated protein expression. (A) SDS-PAGE analysis of Mce-truncated protein expression level with different IPTG concentration at 200 C in *E*. *coli* strain BL21 (DE3) pLysS. (B) SDS-PAGE analysis of Mce-truncated protein production at different time intervals on the optimized concentration of IPTG (1.5mM IPTG). Lane M. Molecular weight marker (MBT092-100LN Hi-Media). Arrow indicates Mce-truncated protein.

**Fig 4:**
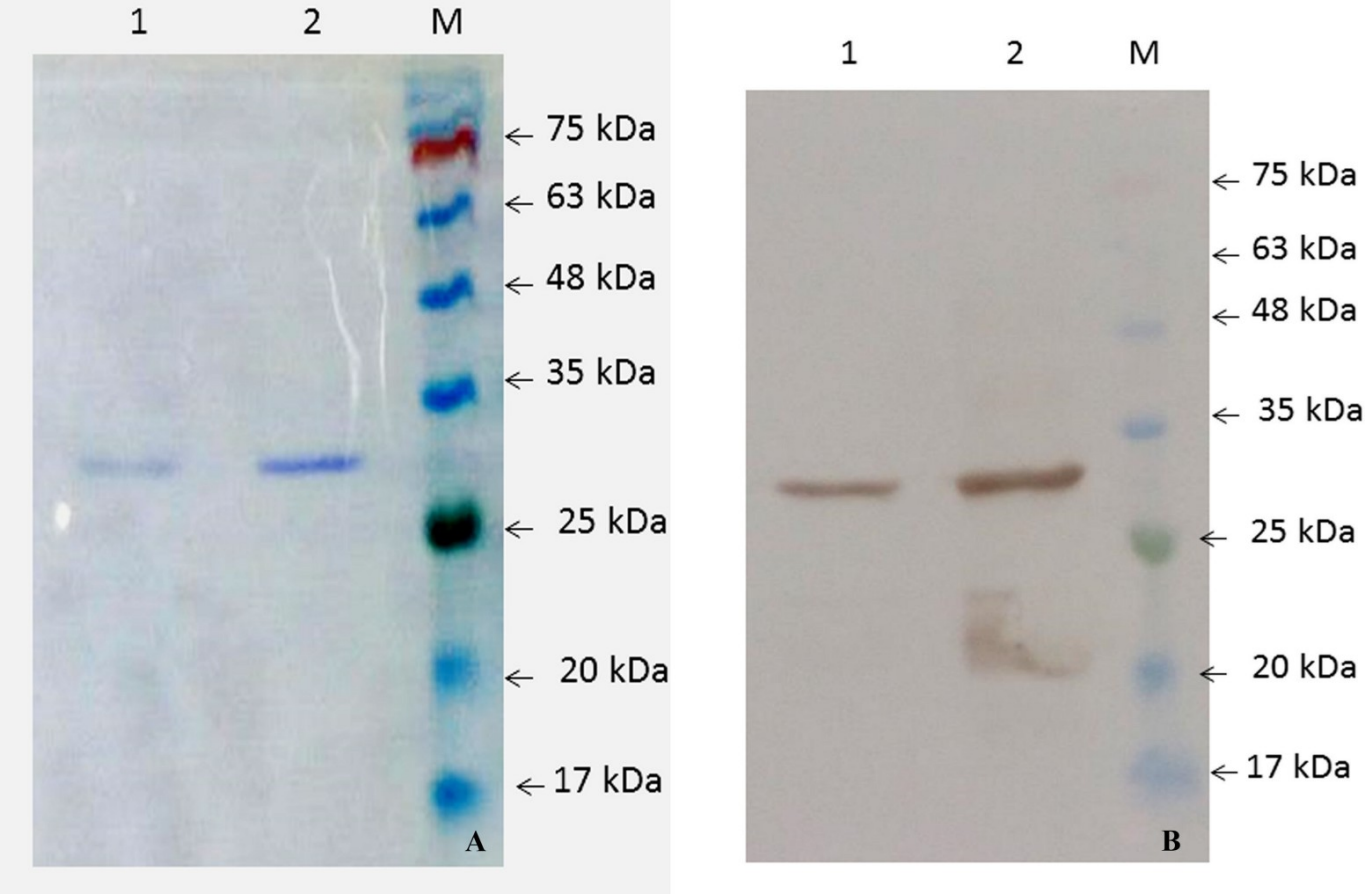
Analysis of purified Mce-truncated protein expressed in *E*. *coli* strain BL21 (DE3) pLysS. (A) SDS PAGE. (Left to Right), Lane 1. pET28a.Mce.truncated Elution 2; lane 2. pET28a.Mce.truncated Elution 1; Lane M. Protein molecular weight marker (SL7012, CinnaGen). (B) Western blot analysis using Anti-6X His-tagged monoclonal antibody (left to right) Lanes 1: and 2: Western blot from purified MAP Mce-truncated protein, Lane M: Protein molecular weight marker (SL7012, CinnaGen).

The pET28a-*mce*-truncated plasmid was transformed into Rosetta/BL-21 cells. Transformed cells were induced for expression with optimized concentration of 1.5 mM IPTG (Fig 3A). Samples were collected at different time intervals and were analyzed using 12.0% SDS-PAGE, along with the protein ladder (Precision plus make). The ~28–30 kDa Mce-truncated protein was expressed from 2 hrs post induction, while zero hour sample and mock controls showed no observable expression; distinct bands of intensity increased with time between 2 and 24 hrs as shown in Fig 3B. Optimized time interval for the best expression of Mce-truncated protein after induction with IPTG was 20 hrs. Expressed proteins were purified by nickel-nitrilotriacetic acid (Ni-NTA) gel matrix (Qiagen, USA) using batch method; purified Mce-truncated protein run on SDS-PAGE showed single band with an approximate expected molecular mass of ~28–30 kDa (Fig 4A). Mce-truncated proteins were confirmed using anti-His tagged mouse monoclonal antibody (Sigma Aldrich, Inc.) (Fig 4B). Final yield of pure Mce-truncated protein per liter for *E*. *coli* culture was approximately 0.8 gr/L using Bradford method.

**Fig 5:**
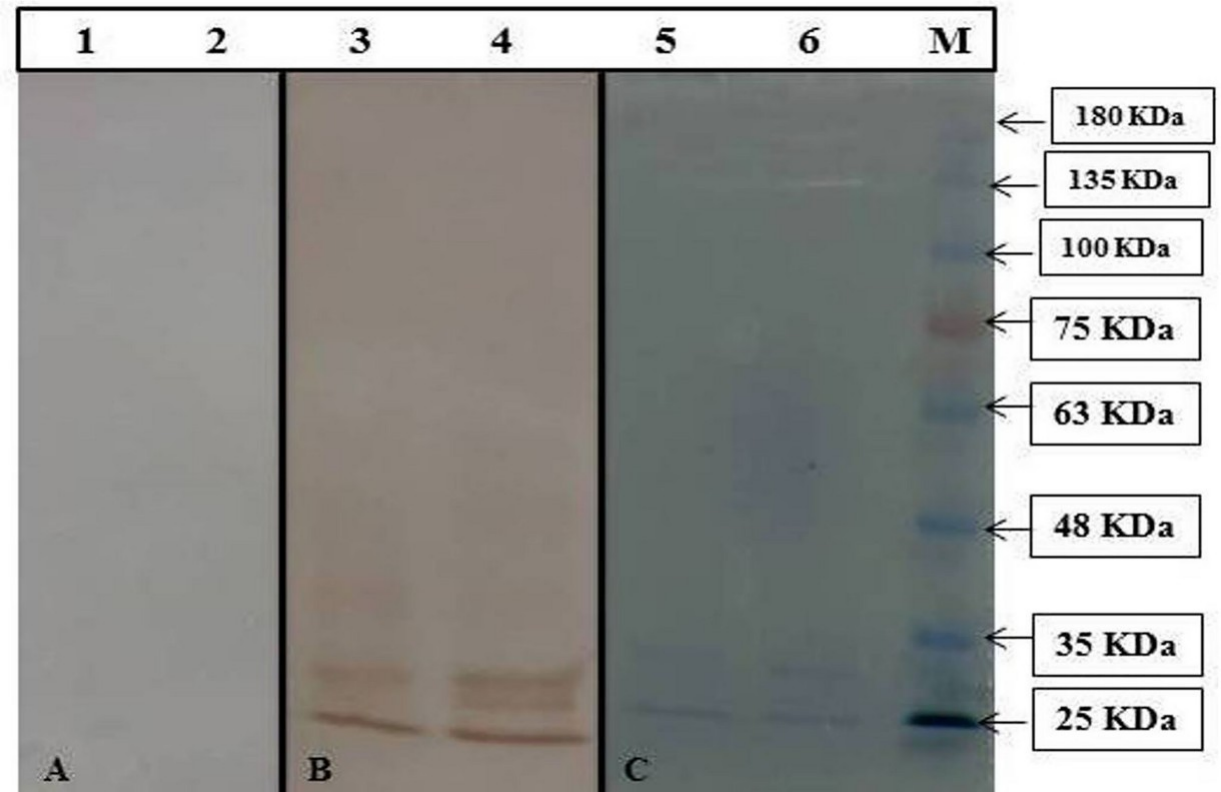
Analysis of cross reactivity and immunogenicity of purified Mce-truncated protein. (A) Western blot analysis using HRP-conjugated anti-bovine immunoglobulin. (Left to Right), Lanes 1, 2. Western blot of Mce-truncated protein of different batch with tuberculin positive sera. (B) Western blot analysis using positive goat serum for MAP. (Left to Right), Lanes 3, 4. Western blot from Mce-truncated proteins of different batch reacted with MAP infected goat serum. (C) SDS PAGE of purified Mce-truncated proteins: Lanes 5, 6. Purified Mce-truncated proteins of different batch, Lane M: Protein molecular weight marker (SL7012, CinnaGen).

Cross reactions analysis by western blot also showed that the expressed Mce-truncated protein reacted with sera of MAP infected animals, but did not react with tuberculosis-infected ones (Fig 5). The result suggested that the recombinant Mce-truncated protein had no cross reactions with non-MAP mycobacterial infections, such as tuberculosis.

### Antibody response with Mce-ELISA

Newly developed Mce-truncated protein based-ELISA was compared with i-ELISA (an in house ELISA using a semi-purified protoplasmic extract of the S5 'Indian Bison' MAP bio-type) (Table 3). Sample OD values (at 450 nm) were converted to sample-to-positive (S/P) ratios as per Collins (2002) [40]. Serum samples in the positive and strong positive categories were considered positive for MAP infection (Table 3). Serum samples from 251 goats, 53 sheep, 117 buffalos, and 33 cattle were screened by Mce-ELISA and i-ELISA tests. Antibody titers measured were 49.4, 51.0, 69.2 and 54.6%, by Mce-ELISA and 55.4, 58.5, 71.8 and 63.6%, by i-ELISA in goats, sheep, buffaloes, and cattle, respectively. Mce-truncated antigen had a higher specificity, but its sensitivity was lower than i-ELISA. However, absorbance values in Mce-ELISA were higher as compared to the values in i-ELISA.

**Table 3:** Comparison of species-wise status of Johne’s disease in ‘Indigenous ELISA’ and ‘Mce ELISA’ tests.

Sera samples (n)	Status	Mce-ELISA (%)	Results (%)	i-ELISA (%)	Results (%)
**Goats (251)**	N	81(32.3)	Negative 127 (50.6%)	65 (25.9)	Negative 112 (44.6%)
	S	9 (3.5)		12 (4.8)	
	LP	37 (14.7)		35 (13.9)	
	P	102 (40.6)	Positive 124 (49.4%)	124 (49.4)	Positive 139 (55.4%)
	SP	22 (8.8)		15 (6)	
**Sheep (53)**	N	19 (35.8)	Negative 26 (49%)	14 (26.4)	Negative 22 (41.5%)
	S	5 (9.4)		6 (11.3)	
	LP	2 (3.8)		2 (3.8)	
	P	23 (43.4)	Positive 27 (51%)	25 (47.2)	Positive 31 (58.5%)
	SP	4 (7.5)		6 (11.3)	
**Buffaloes (117)**	N	22 (18.8)	Negative 36 (30.8%)	10 (8.6)	Negative 33 (28.2%)
	S	2 (1.7)		8 (6.8)	
	LP	12 (10.3)		15 (12.8)	
	P	61 (52.1)	Positive 81 (69.2%)	65 (55.6)	Positive 84 (71.8%)
	SP	20 (17.1)		19 (16.2)	
**Cattle (33)**	N	12 (36.4)	Negative 15 (45.5%)	7 (21.2)	Negative 12 (36.4%)
	S	0 (0)		1 (3.1)	
	LP	3 (9.1)		4 (12.1)	
	P	13 (39.4)	Positive 18 (54.5%)	17 (51.5)	Positive 21 (63.6%)
	SP	5 (15.1)		4 (12.1)	

#### Goats

Of the 251 serum screened by Mce-ELISA and i-ELISA, 124 (49.4%) and 139 (55.4%) were positive, and 127 (50.6%) and 112 (44.6%) were negative, respectively (Table 3). Comparative sero-reactivity of Mce-ELISA with i-ELISA showed that results of positive and negative goats were comparable. A total of 21 (8.3%) positive samples in i-ELISA were missed by Mce-ELISA, while 6 (2.3%) samples-positive in Mce-ELISA were missed by i-ELISA (Table 4). Though i-ELISA appeared to be more sensitive of the two tests applied, but Mce-ELISA was more specific than i-ELISA (Table 5).

**Table 4:** Comparison of two ELISA test (Mce-ELISA and i-ELISA) combinations for the diagnosis of Johne’s disease in domestic livestock species.

Tests	Combinations, *n* (%)			
	1	2	3	4
**Mce-ELISA**	+	-	+	-
**i-ELISA**	+	-	-	+
Goats (251)	118 (47.0)	106 (42.2)	6 (2.3)	21 (8.3)
Sheep (53)	27 (50.9)	22 (41.5)	0 (0.0)	4 (7.5)
Buffaloes (117)	78 (66.6)	30 (25.6)	3 (2.5)	6 (5.0)
Cattle (33)	18 (54.5)	12 (36.3)	0 (0.0)	3 (9.0)

*Value in parenthesis are percent.

**Table 5:** Comparative sensitivity and specificity of two ELISA tests (Mce-ELISA with i-ELISA) in four domestic livestock species.

Species	Tests	TP	TN	FP	FN	Sen & Sp (%)
Goats (251)	Mce-ELISA	118	106	6	21	84.8 & 94.6
	i-ELISA	118	106	21	6	95.1 & 83.4
Sheep (53)	Mce-ELISA	27	22	0	4	87.1 & 100.0
	i-ELISA	27	22	4	0	100.0 & 84.6
Buffaloes (117)	Mce-ELISA	78	30	3	6	92.8 & 90.0
	i-ELISA	78	30	6	3	96.3 & 83.3
Cattle (33)	Mce-ELISA	18	12	0	3	85.7 & 100.0
	i-ELISA	18	12	3	0	100.0 & 80.0

TP- true positive; FP-false positive; TN- true negative; FN- false negative; Sen- sensitivity; Sp- specificity.

#### Sheep

Of the 53 serum screened by Mce-ELISA and i-ELISA, 27 (51.0%) and 31 (58.5%) were positive and 26 (49.0%) and 22 (41.5%) were negative, respectively (Table 3). I-ELISA showed higher number of serum samples positive and lower number as negatives than Mce-ELISA. Only 4 (7.5%) positive samples in i-ELISA were missed by Mce-ELISA (Table 4). Here too, i-ELISA was more sensitive, though specificity was lower than Mce-ELISA (Table 5).

#### Buffaloes

Of 117 buffaloes serum samples screened by Mce-ELISA and i-ELISA, 81 (69.2%) and 84 (71.8%) were positive and 36 (30.8) and 33 (28.2%) were negative, respectively (Table 3). Results were comparable; only 3 (2.5%) positive serum in i-ELISA were missed by Mce-ELISA (Table 4). These results showed higher specificity and lowered sensitivity in case of Mce-ELISA as compared to i-ELISA in buffaloes (Table 5).

#### Cattle

Of 33 cattle serum screened, 18 (54.5%) and 21 (63.6%) were positive, and 15 (45.5%) and 12 (36.4%) were negative by Mce-ELISA and i-ELISA, respectively (Table 3). The 3 (9.0%) serum positive in i-ELISA were missed by Mce-ELISA (Table 4). The results were similar in cattle and Mce-ELISA showed higher specificity and lowered sensitivity in comparison to i-ELISA (Table 5).

#### Sensitivity and specificity of Mce-ELISA

*(a) Comparison of Mce-ELISA with i-ELISA*. Sensitivity of Mce-ELISA was lower 84.8, 87.1, 92.8 and 85.7%, but specificity was higher 94.6%, 100.0%, 90.0 and 100.0% in goats, sheep, buffaloes, and cattle, respectively (Table 5).

*(b) Comparison of Mce-ELISA with i-ELISA*. Sensitivity of i-ELISA was higher 95.1, 100.0, 96.3 and 100.0%, but specificity was lower 83.4%, 84.6%, 83.3 and 80.0% in goats, sheep, buffaloes and cattle, respectively (Table 5).

### Statistical analysis

Mc-Nemar test and kappa agreement were used for statistical analysis. P value and kappa agreement were calculated. P values were 0.0071, 0.1336, 0.5050 and 0.2482, and Kappa agreements were 0.785, 0.849, 0.815 and 0.814 in goats, sheep, cattle, and buffaloes, respectively. The strength of agreement was substantial in goats and perfect in another three livestock species (sheep, buffaloes, cattle), respectively (Table 6).

**Table 6:** P values (McNemar test) and Kappa agreement between Mce-ELISA and i-ELISA.

Species	P values		Kappa	Strength of agreement	95% Confidence interval
	Status	Value			
Goats	Very significantly different	0.0071	0.785	Substantial	0.709 to 0.861
Sheep	Not significantly different	0.1336	0.849	Perfect	0.708 to 0.990
Buffaloes	Not significantly different	0.5050	0.815	Perfect	0.700 to 0.931
Cattle	Not significantly different	0.2482	0.814	Perfect	0.616 to 1.000

Kappa value (0.0–0.20, poor; 0.21–0.40, fair; 0.41–0.60, moderate; 0.61–0.80, substantial and 0.81–100, perfect).

## Discussion

MAP, the cause of JD in domestic livestock [41], has also been associated with CD and other auto-immune disorders in human beings [2, 4, 5, 8]. Developing effective strategies for the diagnosis and control of MAP infection remains a major challenge for optimizing the animal health [42]. ELISA test being sensitive, quick and cost effective is preferred as ‘screening test’ in chronic infections like MAP [37]. Present study described genetic expression of a new Mce protein that could be possibly used as potential antigenic candidate for the development of a new ELISA test for the diagnosis of MAP infection.

Proteins of Mce-family of pathogenic mycobacteria have been associated with virulence by enhancing invasive power and survival in macrophages and various mammalian cell lines [15, 43–46]. Mce encoded products were found to conferring invasive attribute to otherwise a non-pathogenic strain of *E*. *coli*, invading non-phagocytic HeLa cells [16, 24, 47]. Kumar et al. [48] analyzed and compared the expression profiles of the *mce* operons of *Mycobacterium tuberculosis*, *Mycobacterium bovis*, and *Mycobacterium smegmatis* under different growth conditions and found the expression of *mce*1, *mce*3, and *mce*4 operons present in tissues collected from infected guinea pigs and rabbits. Hou et al. [49] found that genes similar to MTB *mce* genes were expressed by *Mycobacterium avium* during growth in macrophages. Another previous study also revealed presence of *mce* and *PE*/ *PPE* family genes in MAP as virulence factors [50]. In a study conducted by Wu et al. [51], showed that there were islands in both *M*. *avium* subsp. *avium* (MAC) and MAP which encode for different *mce* genes that were analogous in all the mycobacteria, indicating their functional importance.

*MAP2191* gene is one of the *mce*5 operons (*MAP2189* to *MAP2194*) of the MAP; *mce*5 cluster which has previously been identified as important for invasion and survival of bacilli within macrophages and also virulence of MAP [25, 26]. Eight putative Mce-encoding genes have been identified in the MAP strain K-10 genome. Some studies have been carried out to find the unique MAP-specific protein targets, which may elicit strong immune responses in the host [13, 52]. Targeting of the *MAP2191* gene was based on the identification of putative B- and T-cell epitopes in MAP proteins encoded by the *mce*5 gene cluster and asses their antigenicity during infection. Using Bioinformatics analyses, we identified B- and T-cell epitopes in the C-terminal region of the *MAP2191* putative Mce protein. The selected region of MAP2191 protein had a high antigenic index and was more hydrophilic as crevealed by the CLC software analysis. In this study, we generated and cloned a truncated *MAP2121* DNA sequence, and subsequently generated the His-tagged recombinant protein in an *E*. *coli* host. Our purification method with Ni-NTA agarose resin was very effective and we obtained a large amount of soluble and purified Mce-truncated peptide.

Currently, antigen-based ELISA tests for detecting MAP with a mixture of proteins are available including avian or johnin, a MAP-PPD, whole-cell sonicated extract, protoplasmic antigens etc., [53]. Because analysis and sequencing of the entire MAP genome has been recorded [54], immune reactivity of several specific proteins of the MAP genome have been investigated [55]. In the present study also, serological ELISA assays were conducted and results have been reported for the new ELISA test developed using truncated MAP Mce protein and the traditional i-ELISA kit.

This in house i-ELISA kit has been validated with other commercial ELISA kits (ID-VET, Allied Monitor Inc., USA, antigen based indirect ELISA, Ethanol Vortex ELISA (EV-ELISA), recombinant secretory proteins based cocktail ELISA (c_ELISA) and natural secretory proteins-based ELISA test [10, 35, 37, 56, 57] and compared its efficacy in different livestock species (goats, sheep, cattle and buffaloes) [12, 58, 59]. In this series, Mce-ELISA is the most recently developed ELISA and used for the diagnosis of MAP infection. I-ELISA has been the most validated and robust test was used for evaluating newly developed Mce-ELISA in terms of specificity and sensitivity.

Our earlier studies reported enhanced sensitivity and specificity of the i-ELISA as compared to the other commercially available ELISA kits for the diagnosis of Johne’s disease in domestic livestock [38, 39]. For example, in a study, i-ELISA kit showed greater diagnostic efficiency than other commercial ELISA kits [38] and improved the detection rate of MAP in test samples [60]. We used S/P-ratios-based categorization of OD values as per Collin (2002), wherein serum samples in the positive and strong positives categories, were considered as positive for MAP infection. This type of categorization exhibited good correlation with fecal culture in some of our other studies [39, 61]. Chaubey *et al*. (2015) compared i-ELISA (Indigenous g-ELISA) with commercial antigen-based b-ELISA and commercial sr-ELISA and found 100% sensitivity. In another report, 91.4% sensitivity of i-ELISA was observed when compared with EV-ELISA kit [57]. These and other findings from other studies confirmed the enhanced sensitivity and specificity of i-ELISA in the detecting of MAP infection in domestic livestock in the country [35, 38].

In our study, Mce-truncated protein yielded higher absorbance values against the confirmed positive pooled serum samples, and low-level (but above background) antibody reactivity was also observed with confirmed negative pooled serum samples. The ELISA test using Mce-truncated protein was able to discriminate between the most positive and negative serum samples used in this study. However, the Mce-based ELISA showed a relatively higher specificity than i-ELISA but suffered from slight reduction in sensitivity. These results highlighted the potential capability of Mce-truncated ELISA in preventing false-positive reactions that occur when a milieu of antigens is used in i-ELISA tests. Mce-ELISA has shown perfect agreement with i-ELISA and there is no cross-reactivity with anti-*M*.*bovis* antibodies. Therefore, this test could be a better choice for screening of paratuberculosis especially in the region where bovine-TB is endemic. Overall the Mce-based ELISA was comparable to the the highly sensitive and robust ‘i-ELISA test’, indigenously developed for the screening of livestock population.

In conclusion, this is the first report of a Mce surface protein-based ELISA as new modified ELISA test, as diagnostic test against MAP infection. Affinity of purified Mce-truncated protein was detected by serum antibodies from animals infected with JD and supported the hypothesis that MAP Mce protein was immunogenic and highly expressed protein during MAP infection. True achievement of this study was the isolation and expreseeion of the Mce protein, ‘a new antigen’ with the huge potential to be further exploited not only as a diagnostic tool but also a new target antigen for the development of a highly effective vaccine for the control and eradication of the incurable Johne’s disease in domestic livestock.

## Supporting information

S1 Appendix (DOCX)

S1 Raw Images (DOCX)
