# Supplementary material for: Novel recombinant Mce-truncated protein based ELISA for the diagnosis of Mycobacterium avium subsp. paratuberculosis infection in domestic livestock
Source: PLoS One. 2020 Jun 1;15(6):e0233695. doi: 10.1371/journal.pone.0233695 (PMC7263793; doi:10.1371/journal.pone.0233695)
Supplement: S1 Appendix — (DOCX) [file pone.0233695.s001.docx]

**Table 1S.** Bacterial Strains

| S. No. | Strain | Genotype | Source/ Reference |
| --- | --- | --- | --- |
| 1. | XL10-Gold Ultracompetent Cells | TetrD(mcrA)183 D(mcrCB-hsdSMR-mrr)173 endA1 supE44 thi-1 recA1 gyrA96 relA1 lac Hte [F´ proAB lacIqZDM15 Tn10 (Tetr) Amy Camr] | Stratagene, Agilent |
| 2. | Rosetta | F^-^ *ompThsdS*_B_(r_B_^-^ m_B_^-^) *gal dcm* (DE3) pRARE (Cam^R^) | Novagene, WI, USA |
| 3. | Bl-21 | F–ompT hsdS(rB–mB ­) gal dcm λ(DE3) pLysS (Camr) (λ(DE3): lacI, lacUV5-T7 gene 1, ind1, sam7, nin5) | Novagene, WI, USA |
| 4. | DH5α | F-80dlacZ M15 (lacZYA-argF) U169 recA1 endA1hsdR17(rk-, mk+) phoAsupE44 -thi-1 gyrA96 relA1 | Invitrogen, USA |

**Table 2S.** Cloning and expression vector, kits and restriction enzymes

| MAP *mce* Gene | TA Cloning | | Restriction enzyme | Cloning in expression vector | |
| --- | --- | --- | --- | --- | --- |
|  | Vector/ kit | Cells used in Transformation |  | Cloning & expression vector | The cells used in Transformation |
| MAP2191 Truncated gene | pTZ57R/T Cloning vector, InsTAclone PCR Cloning Kit (#1214) | DH5α & XL-10 Ultracompetent cells | BamHI & HindIII, Thermo scientific, USA | pET28a(+) expression vector, Novagene, Billerica, MA,USA | DH5α & XL-10 Ultracompetent cells |

**Table 3S.** Antibiotics and Supplements

| S. No. | Antibiotics/ Supplements | Final Concentration (µg/ml) | | Supplier |
| --- | --- | --- | --- | --- |
| 1. | Kanamycin | 50 | Amresco (Cat No. 0408-10G) | |
| 2. | Ampicillin | 100 | Amresco (Cat No. 0339-25G) | |
| 3. | Chloramphenicol | 20 | Sigma (Cat No. C6455) | |
| 4. | Tetracycline | 20 | Sigma (Cat No. 87128-25G) | |
| 5. | X-gal^1^* | 20 | Sigma (Cat No. XGAL-RO) | |
| 6. | IPTG^2^* | 20 | Genetix (Cat no. R0392) | |

^1*^ 5-Bromo-4-chloro-3-indolyl-β-D-galactopyranoside

^2*^ Isopropyl-β-D thiogalactopyranoside

**Table 4S.** Primers used for characterization of MAP (Millar *et al*. 1996)

| **Target** | **Primer** | **Primer sequence** | **Product Size** |
| --- | --- | --- | --- |
| IS *900* | Forward: P90 | 5' GAA GGG TGT TCG GGG CCG TCG CTT AGG 3' | **413 bp** |
|  | Reverse: P91 | 5' GGC GTT GAG GTC GAT CGC CCA CGT GAC 3' |  |

**Table 5S.** Primers used for genotyping of MAP (Silleva *et al*., 2005)

| Target | Primer | Primer sequence | Product Size | Restricted size (bp) |
| --- | --- | --- | --- | --- |
| IS*1311* | Forward: M 56 | 5' GCG TGA GGC TCT GTG GTG AA 3' | 608 bp | Sheep: 285, 323 Cattle: 67, 218. 285,323 Bison: 67, 218, 323 |

**Table 6S.** Optimized conditions of expressing Mce-truncated gene

| **Name** | **Expression status** | **Protein status** | **Bacterial expression cell** | **IPTG concentration** | **Expression Condition** |
| --- | --- | --- | --- | --- | --- |
| Mce-  truncated | Expressed | Good expression, soluble (Crude) | BL21 | 1.5 mM | 20 hrs. at 24^◦^C |

**Table 7S.** Optimization of blocking buffer for ELISA test

| Samples | Conc. Of Blocking agent % | Skimmed milk (SM) | | | Bovine Serum Albumin (BSA) | | |
| --- | --- | --- | --- | --- | --- | --- | --- |
|  |  | Mean OD at 450 nm | | Noise ratio | Mean OD at 450 nm | | Noise ratio |
|  |  | Positive Serum | Negative Serum |  | Positive Serum | Negative Serum |  |
| Goats and sheep | 3 | 0.282 | 0.092 | **0.190** | 0.264 | 0.151 | 0.113 |
|  | 5 | 0.273 | 0.120 | 0.153 | 0.266 | 0.133 | 0.133 |
|  | 10 | 0.261 | 0.138 | 0.123 | 0.279 | 0.141 | 0.138 |
| Cattle and Buffalos | 3 | 0.270 | 0.053 | 0.217 | 0.293 | 0.153 | 0.140 |
|  | 5 | 0.281 | 0.051 | **0.228** | 0.286 | 0.137 | 0.156 |
|  | 10 | 0.284 | 0.089 | 0.195 | 0.246 | 0.109 | 0.137 |

### *MAP 2191* whole and truncated gene sequence link:

https://www.ncbi.nlm.nih.gov/nuccore/?term=MAP2191

### MAP 2191 whole and truncated protein sequence link:

https://www.ncbi.nlm.nih.gov/protein/489972741


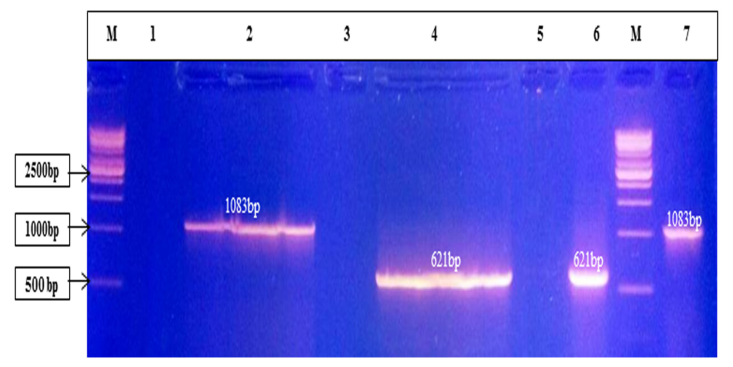


**Fig 1S.** PCR for MAP *mce*-whole and *mce*-truncated genes, lanes M: 1kb DNA ladders (#SM0313, Fermentas), lanes 2: Bulk amplification for MAP *mce*-whole gene, lane 4: Bulk amplification for *mce*-truncated gene; lane 6: Positive by PCR for MAP *mce*-truncated gene; lane 7: positive by PCR for MAP *mce*-whole gene.


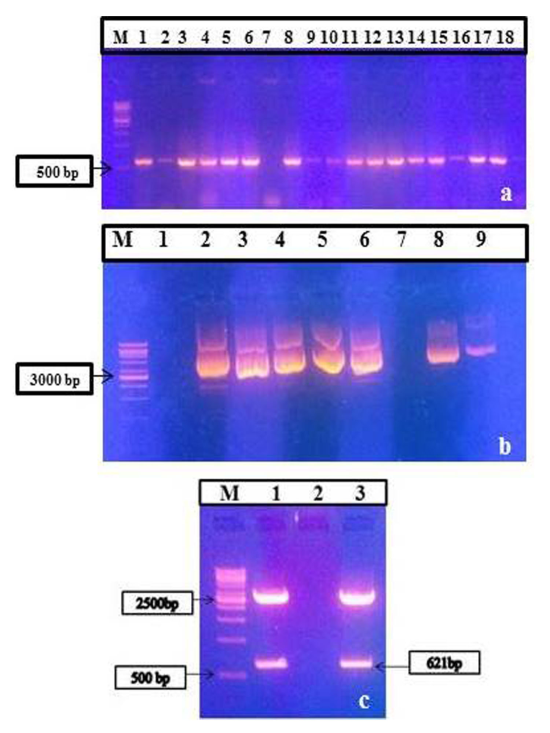


**Fig 2S.** (**a)** Colony PCR of transformed colonies: Lane M: 1kb DNA ladder (Fermentas), Lane 1: Positive control (MAP Indian Bison type DNA), Lane 2: Negative control (NFW), Lanes 3 and 5-11: Positive samples, Lane 4: Negative sample; (**b)** Confirmed transformed plasmid isolation: Lane M: 1 kb DNA ladder (ABC), Lanes 1 and 7: Empty, LaneS 2-6: Truncated *mce*-PTZ57R/T plasmid in DH5α, LaneS 8 and 9: Truncated *mce*-PTZ57R/T plasmid in XL-10; **(c)** Restriction digestion of confirmed PTZ57R/T *mce* /truncated clones: Lane M: 1kb DNA ladder (Fermentas), Lanes 1 and 3: Truncated *mce* gene-PTZ57R/T plasmid, Lane 2: Empty


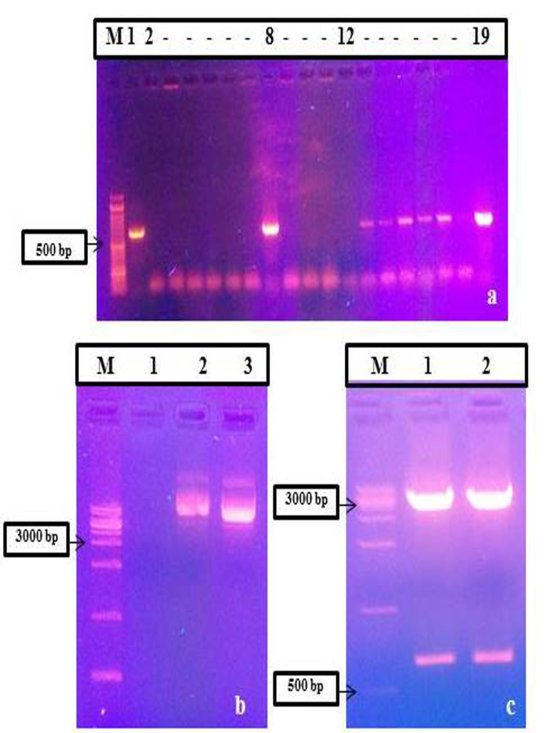


**Fig 3S. (a)** Colony PCR, Lane M. 50bp ladder (Hi-Media), Lane 1: Positive control (MAP Indian Bison type DNA), Lane 2: Negative control (NFW), Lanes 8 and 19: good *mce*/truncated-pET28a clone; **(b)** Plasmid Isolation of confirmed *mce*/truncated-pET28a plasmid, Lane M: 1kb DNA ladder (RBC), Lane2: Empty, Lane 3: *mce*/truncated-pET28a plasmid, Lane 3: pET28a expression vector (Control); **(c)** Restriction Digestion of confirmed clone, Lane M: 1kb DNA ladder (RBC), Lanes 1 and 2: Truncated *mce* gene/ pET28a plasmid


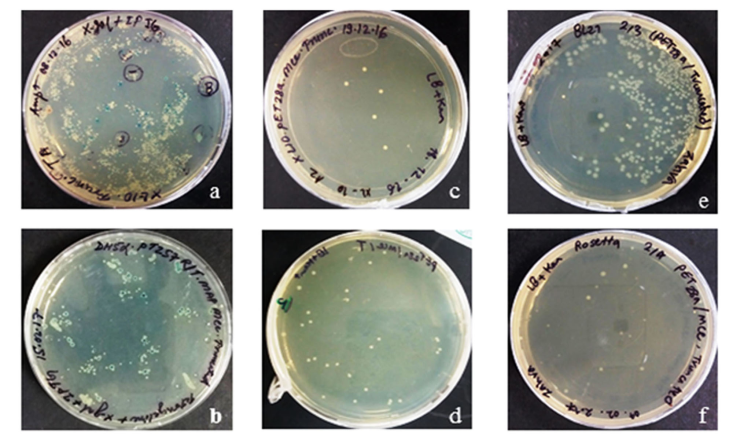


**Fig 4S. (a)** *mce*/truncated-TA Cloning vector in XL-10 cells; **(b)** *mce*/truncated-TA Cloning vector in DH5α cells, (Blue colonies containing normal plasmid without insert and white colonies containing plasmid has insert); **(c)** Colonies containing *mce*/truncated-pET28a plasmid in Xl-10 cells; **(d)** Colonies containing *mce*/truncated-pET28a plasmid in DH5α cells; **(e)** Colonies containing *mce*/truncated-pET28a plasmid in BL21 (DE3) cells; **(f)** Colonies containing *mce*/truncated-pET28a plasmid in Rosetta cells.

**
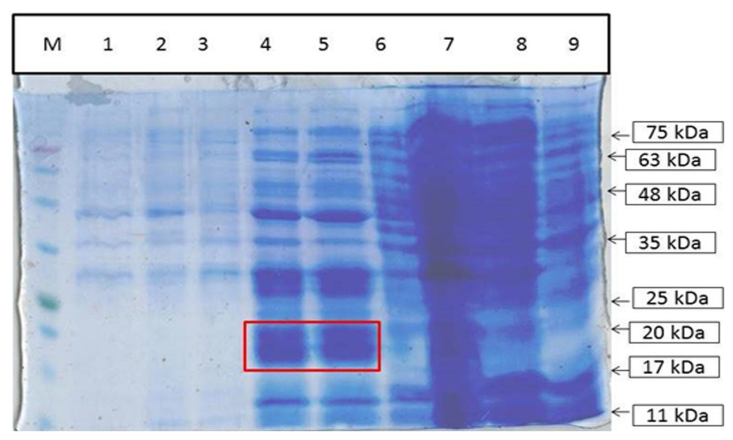
**

**Fig 5S.** Optimization of purification of Mce-truncated protein with ni-NTA beads in BL-21 cells: (left to right). Lane M. Pre-stained page ladder (MBT092-100LN Hi-Media), lane 1. pET28a-Mce-truncated wash 3, lane2. pET28a. Mce-truncated wash 2, lane 3. pET28a-Mce-truncated wash 1, lane 4. pET28a-Mce-truncated Elution 2, lane 5. pET28a- Mce-truncated Elution 1, lane 6. pET28a-Mce-truncated supernatant, lane 7. pET28a-Mce-truncated crude, lane 8. pET28a-Mce-truncated (0.0 mM IPTG): Control, lane 9. BL-21.pET28a

**
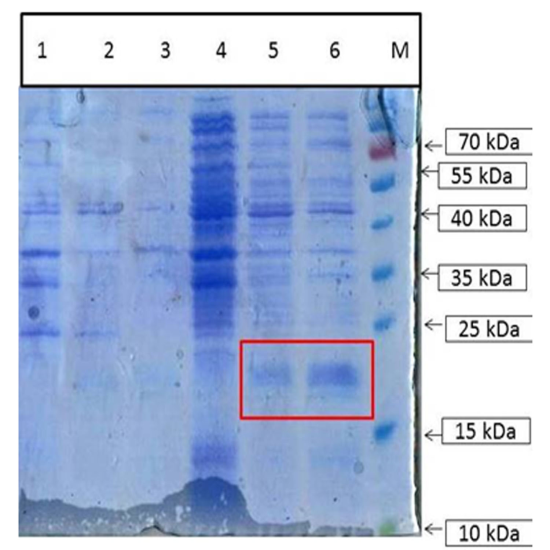
**

**Fig 6S.** Optimization of purification of Mce-truncated protein with ni-NTA beads in Rosetta cells: (left to right). Lane 1: pET28a-Mce-truncated supernatant, lane 2: pET28a-Mce-truncated Elution 2, lane 3: pET28a-Mce-truncated Eloutin 1, lane 4: pET28a-Mce-truncated supernatant, lane 5: pET28a-Mce-truncated. Elution 2, lane 6: pET28a-Mce-truncated Elution 1, lane M. Pre-stained page ladder (#0671, Fermentas)
