# Supplementary material for: Novel recombinant Mce-truncated protein based ELISA for the diagnosis of Mycobacterium avium subsp. paratuberculosis infection in domestic livestock
Source: PLoS One. 2020 Jun 1;15(6):e0233695. doi: 10.1371/journal.pone.0233695 (PMC7263793; doi:10.1371/journal.pone.0233695)
Supplement: S1 Raw Images — (DOCX) [file pone.0233695.s002.docx]

**
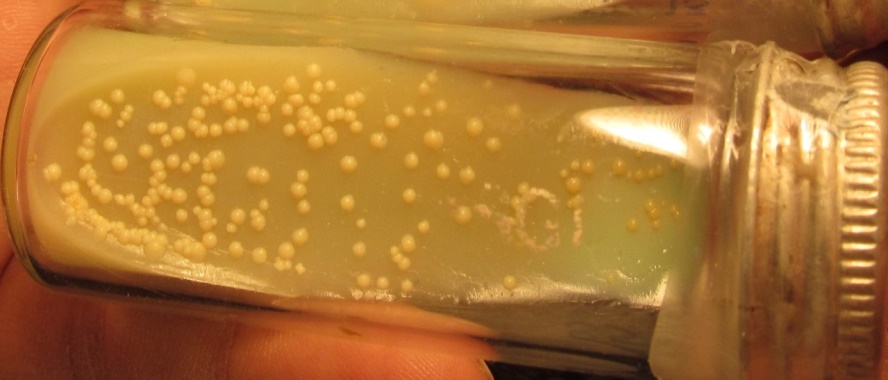
**

**Fig-1A**

**
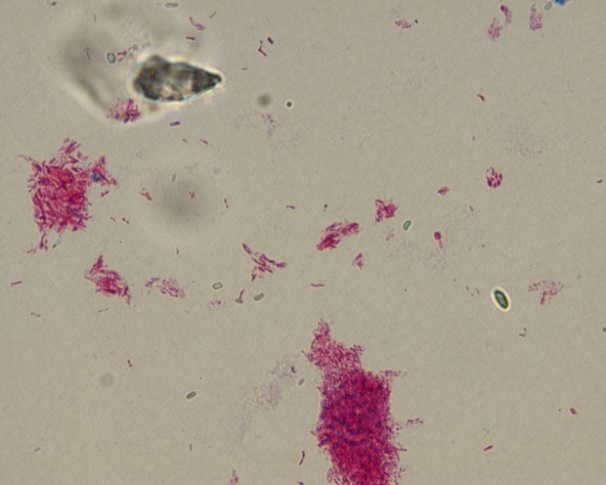
**

**Fig-1B**

**
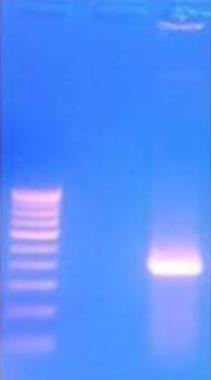
**

**Fig-1C**

**
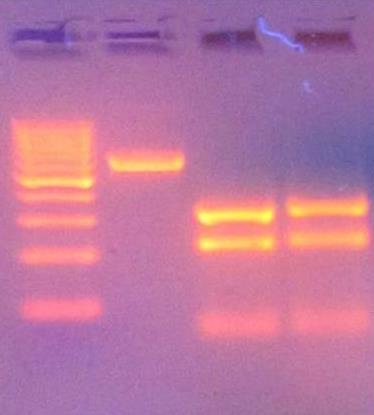
**

**Fig-1D**

**
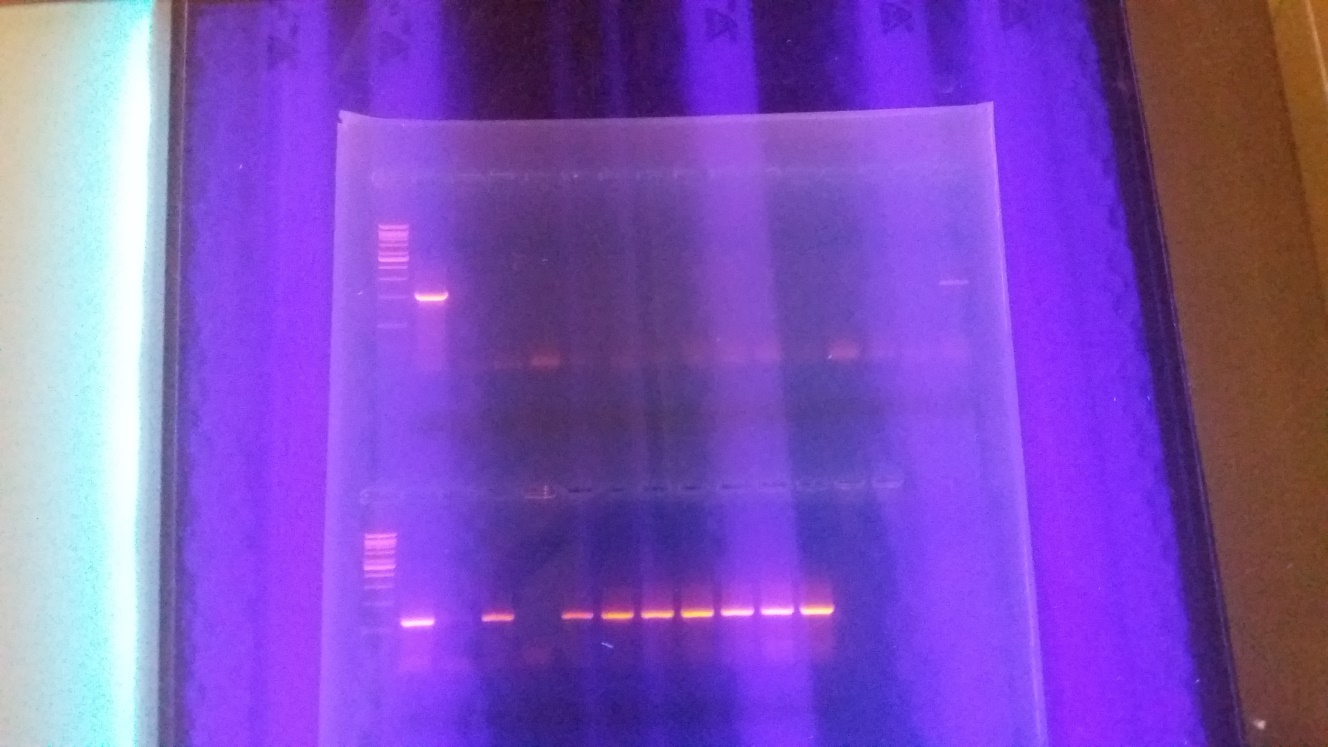
**

**Fig-2A**

**
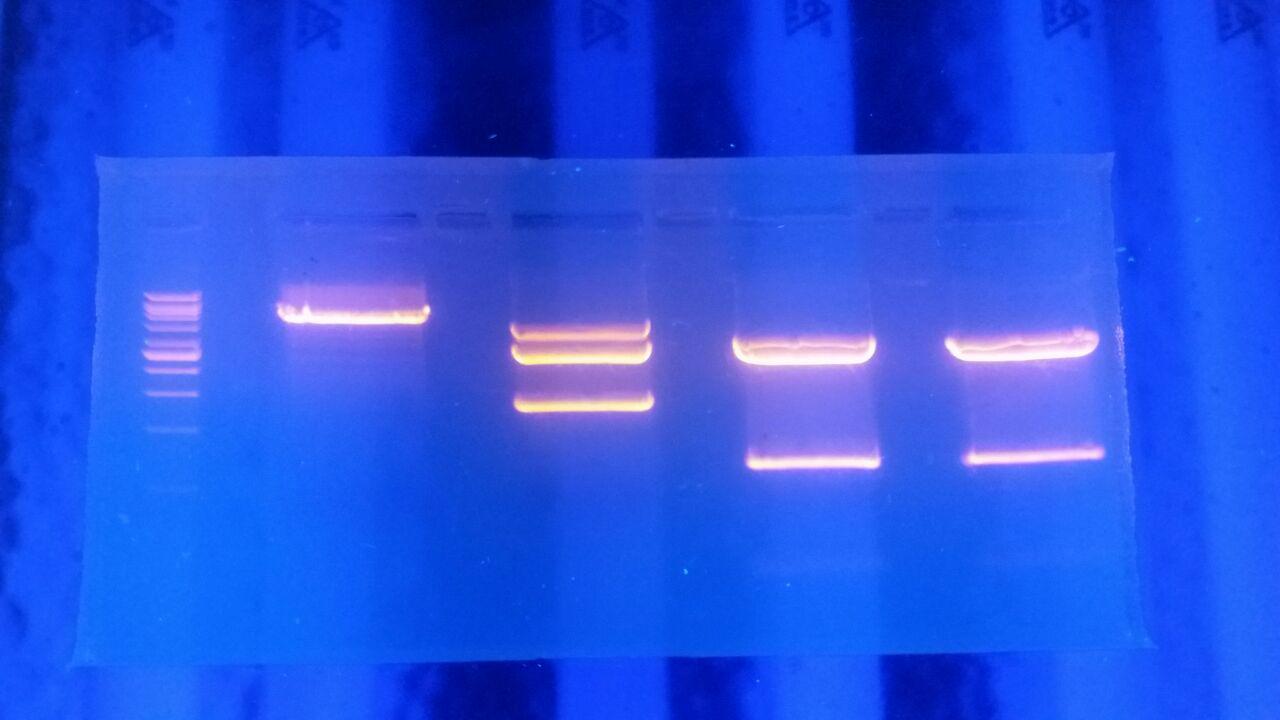
**

**Fig-2B**

**
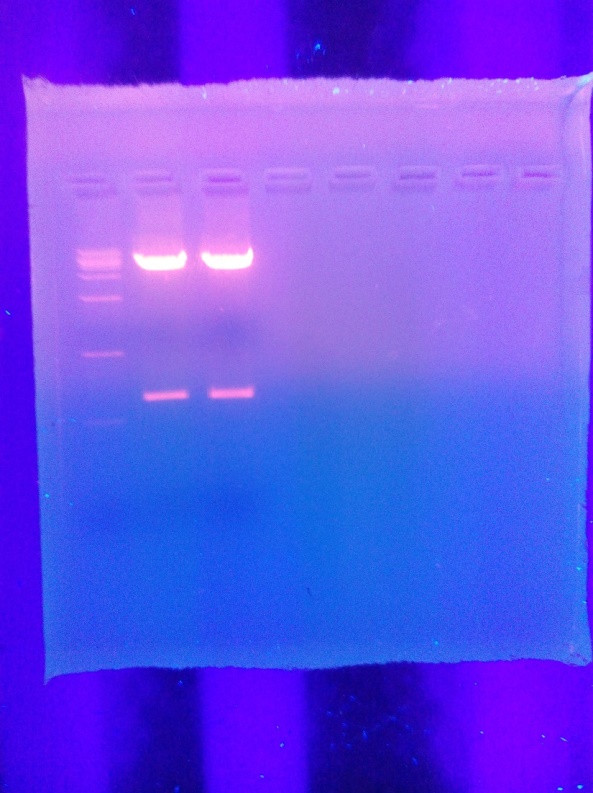
**

**Fig-2C**

**
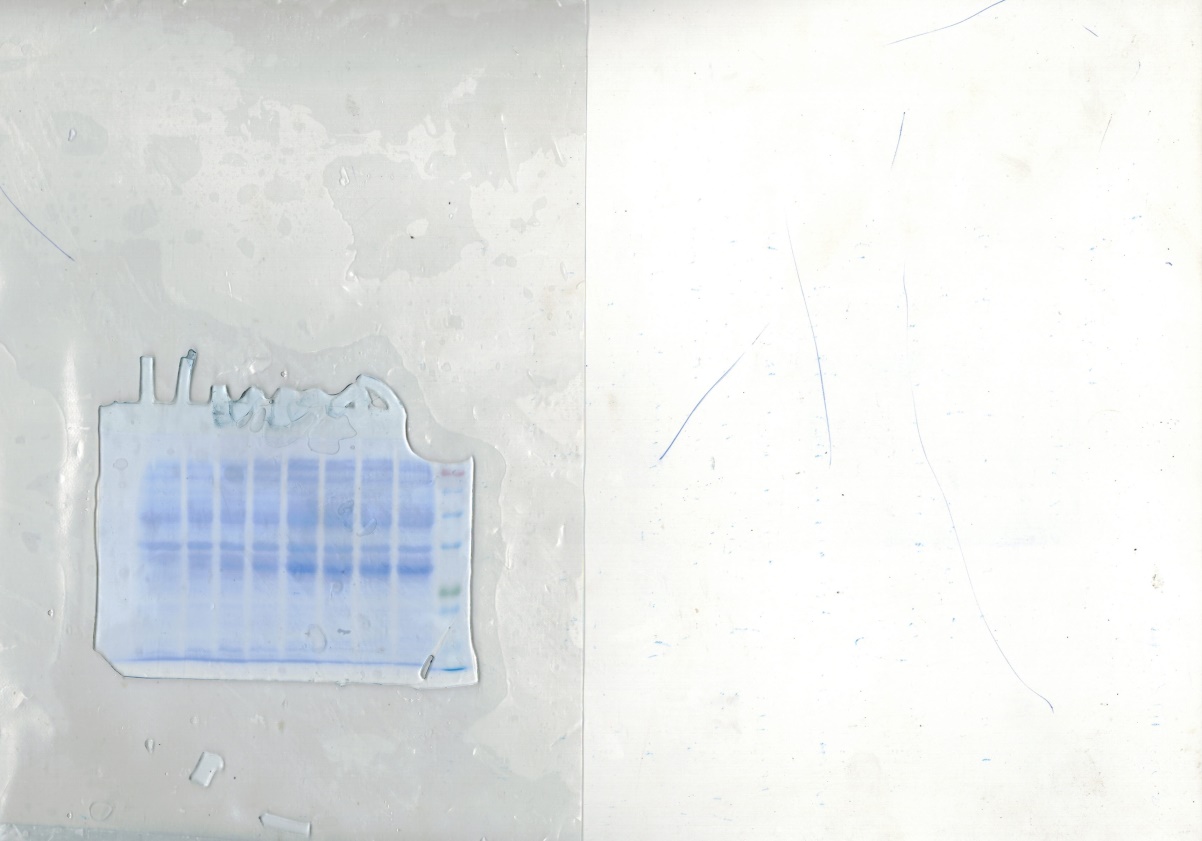
**

**Fig-3A**

**
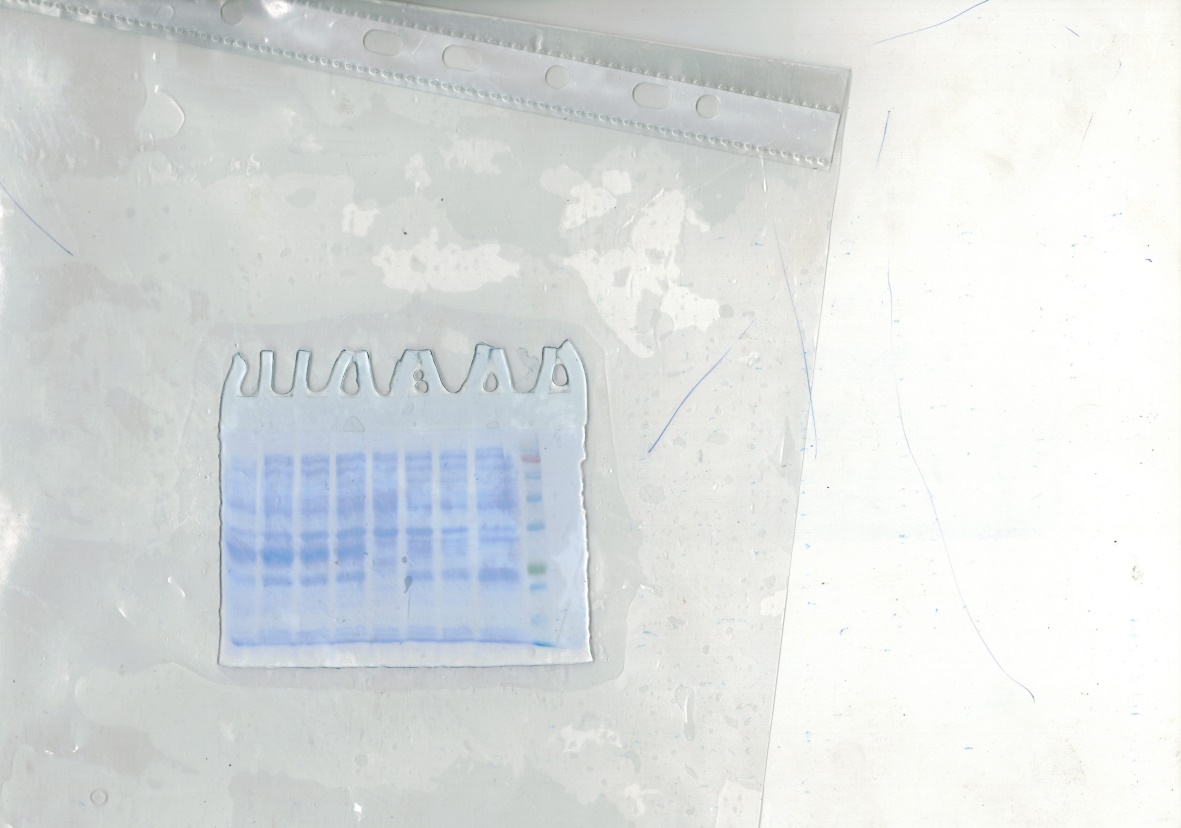
**

**Fig-3B**

**
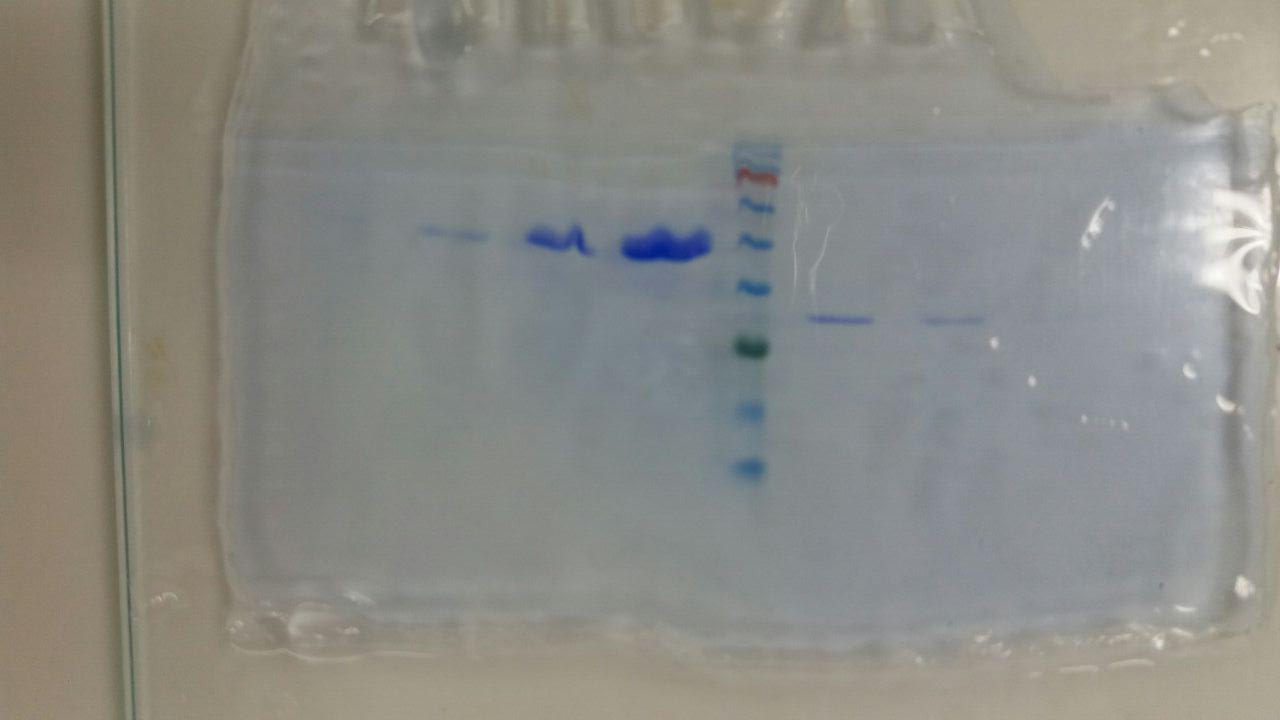
**

**Fig-4A**

**
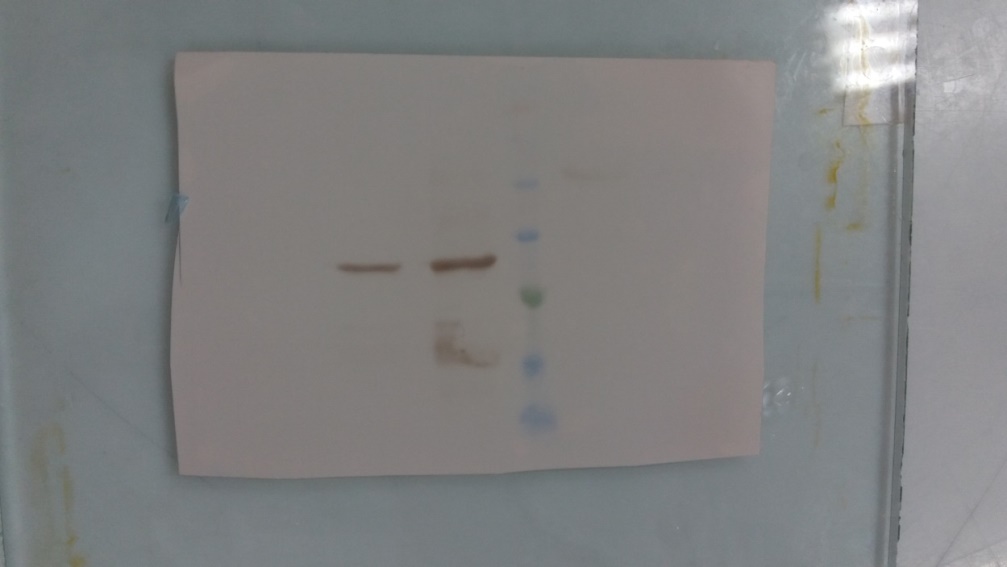
**

**Fig-4B**

**
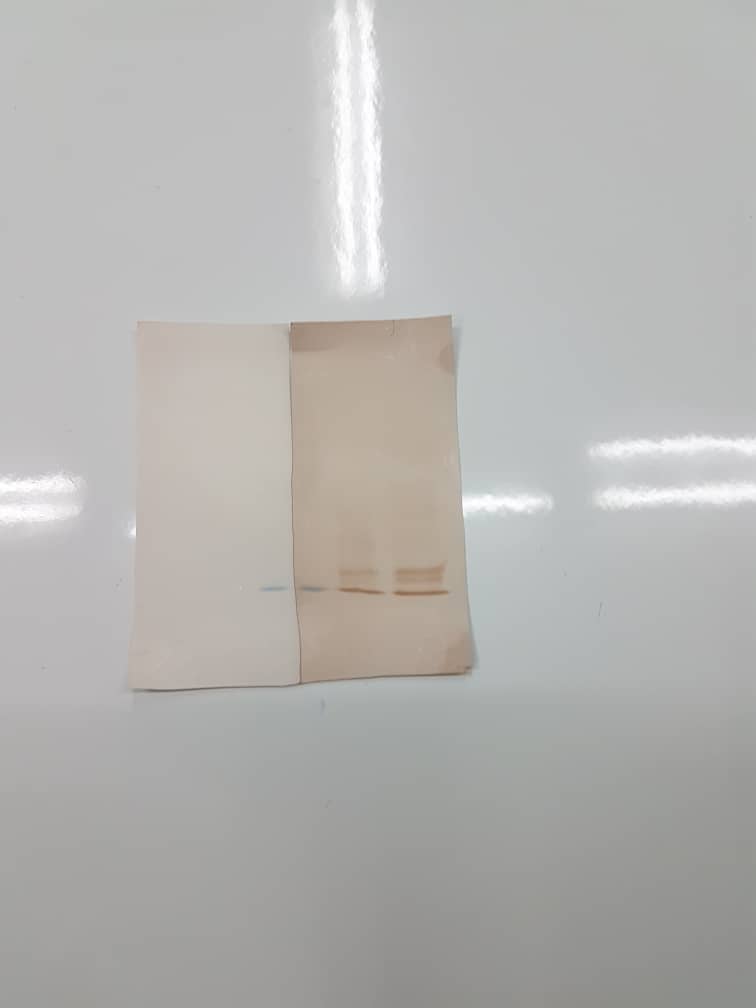
**

**Fig-5A and Fig-5B**

**
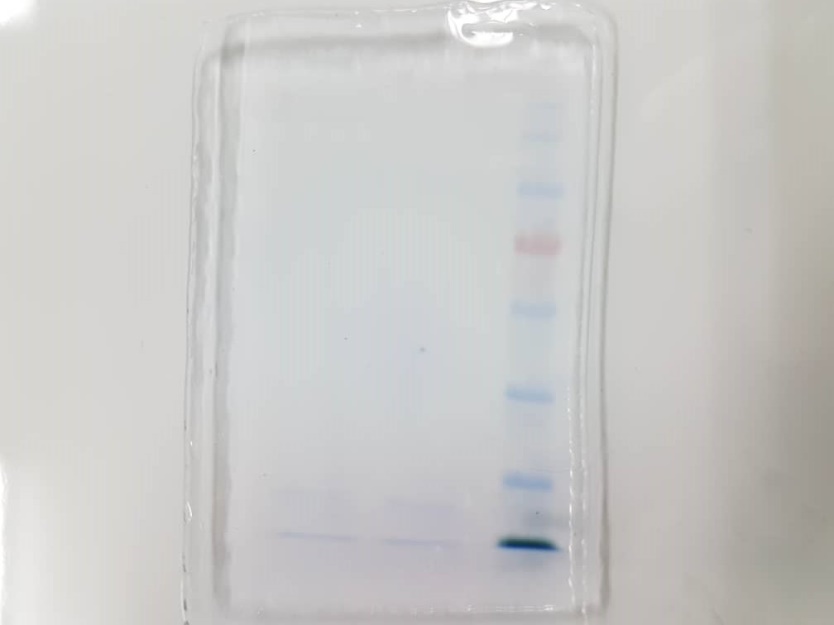
**

**Fig-5C**

**
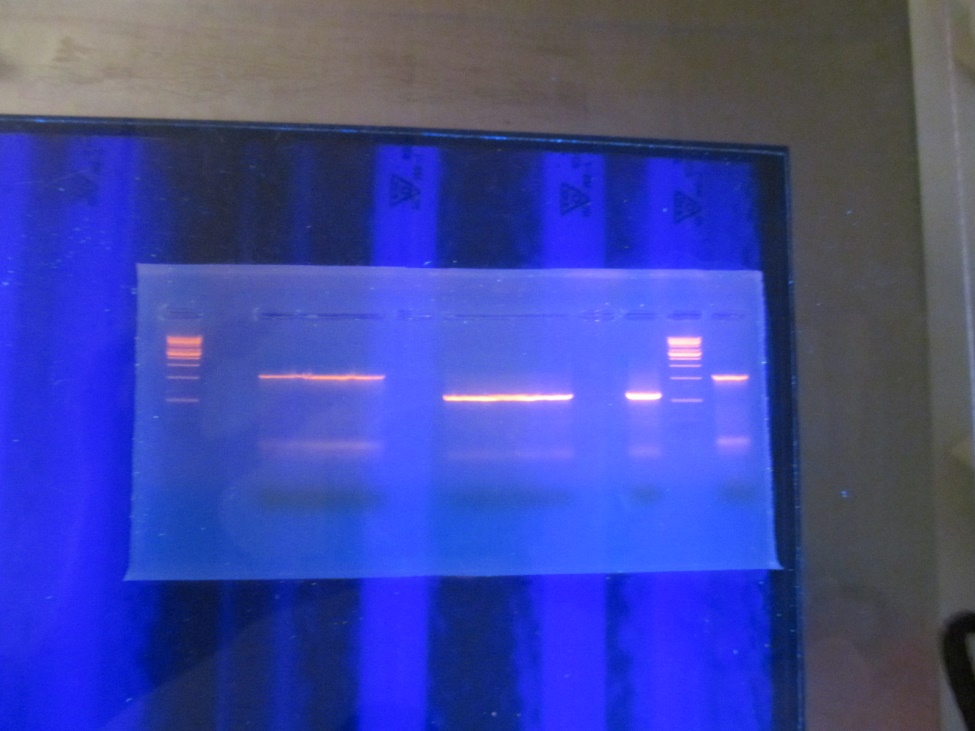
**

**Fig-1S**

**
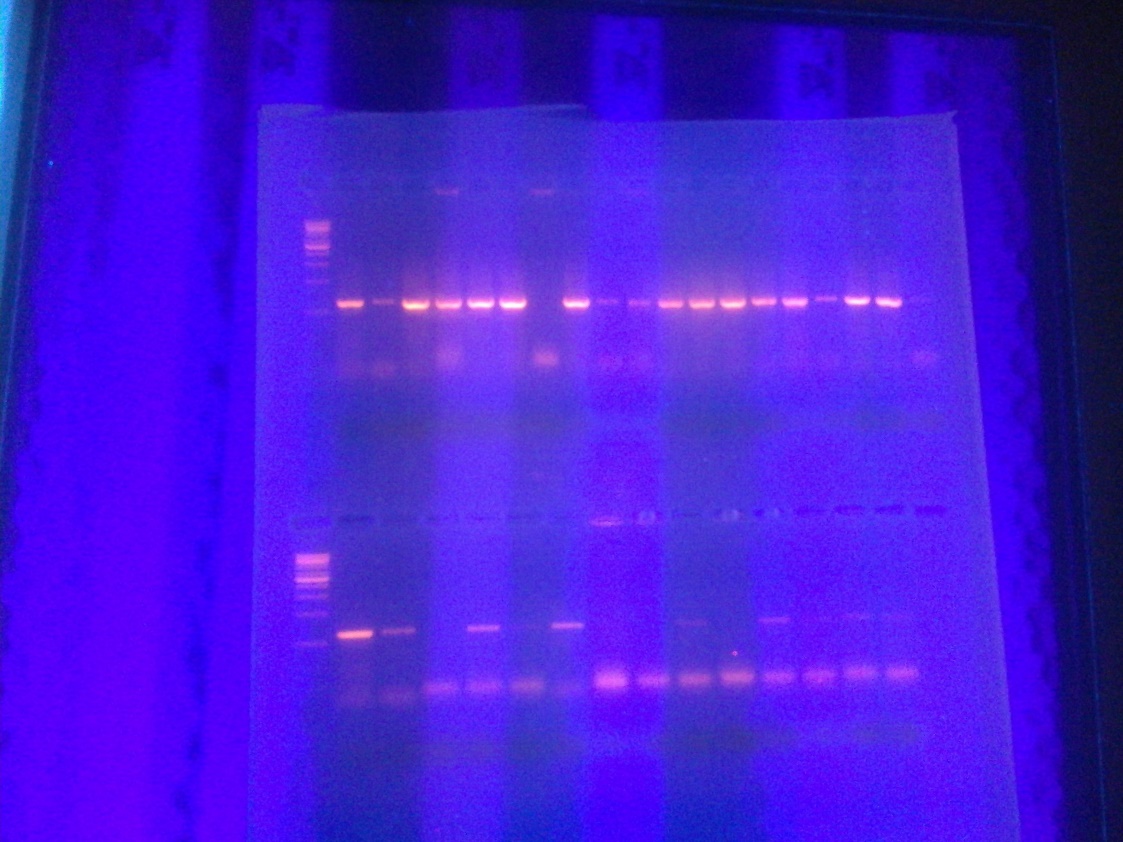
**

**Fig-2S (a)**

**
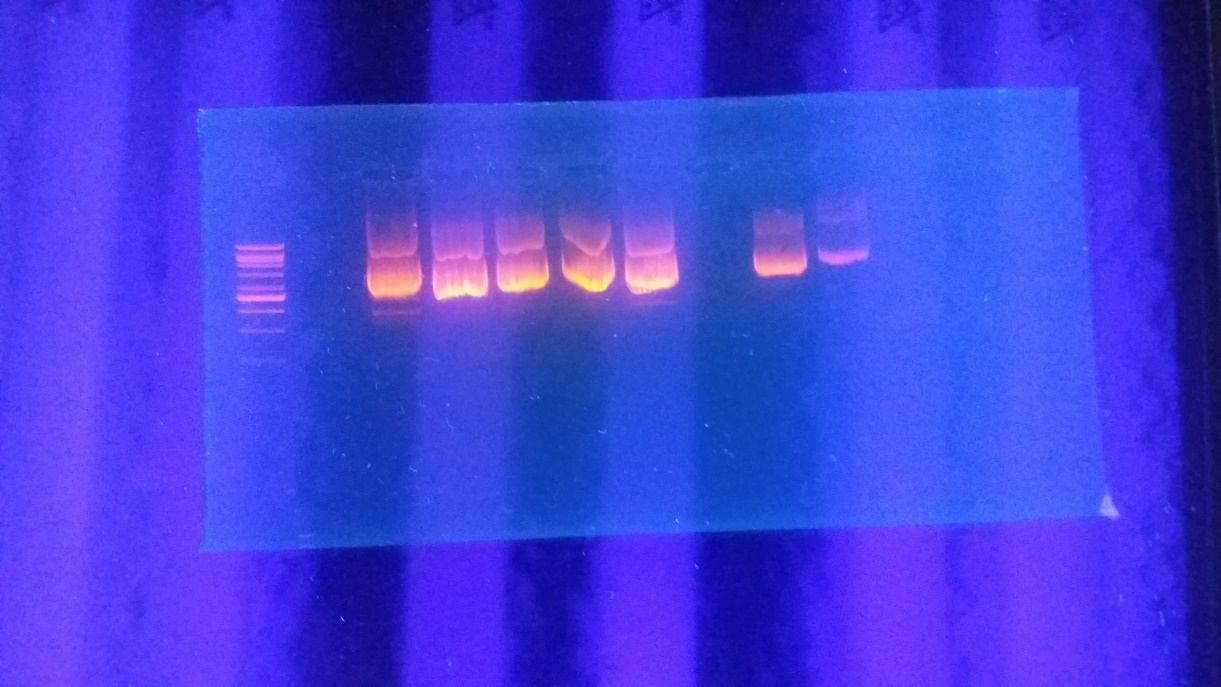
**

**Fig-2S (b)**

**
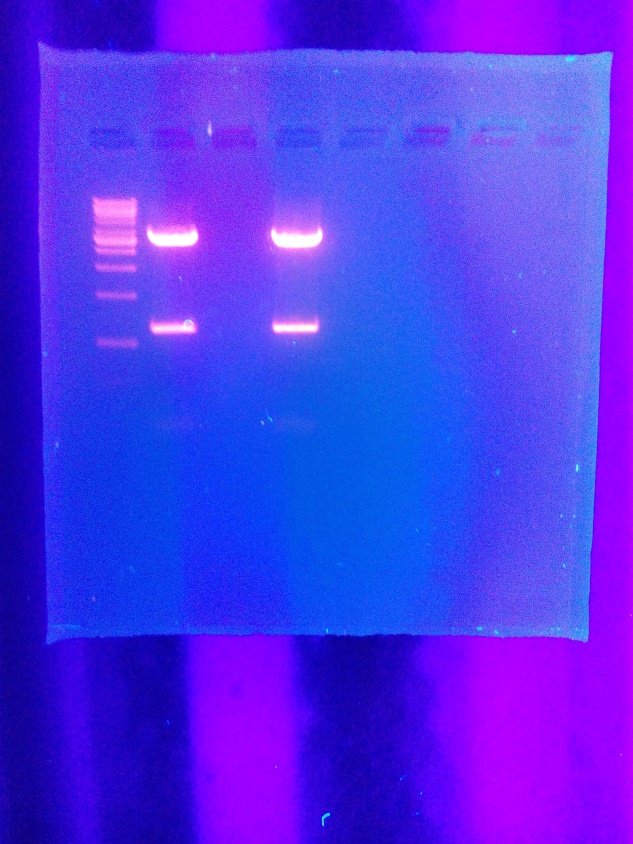
**

**Fig-2S (C)**

**
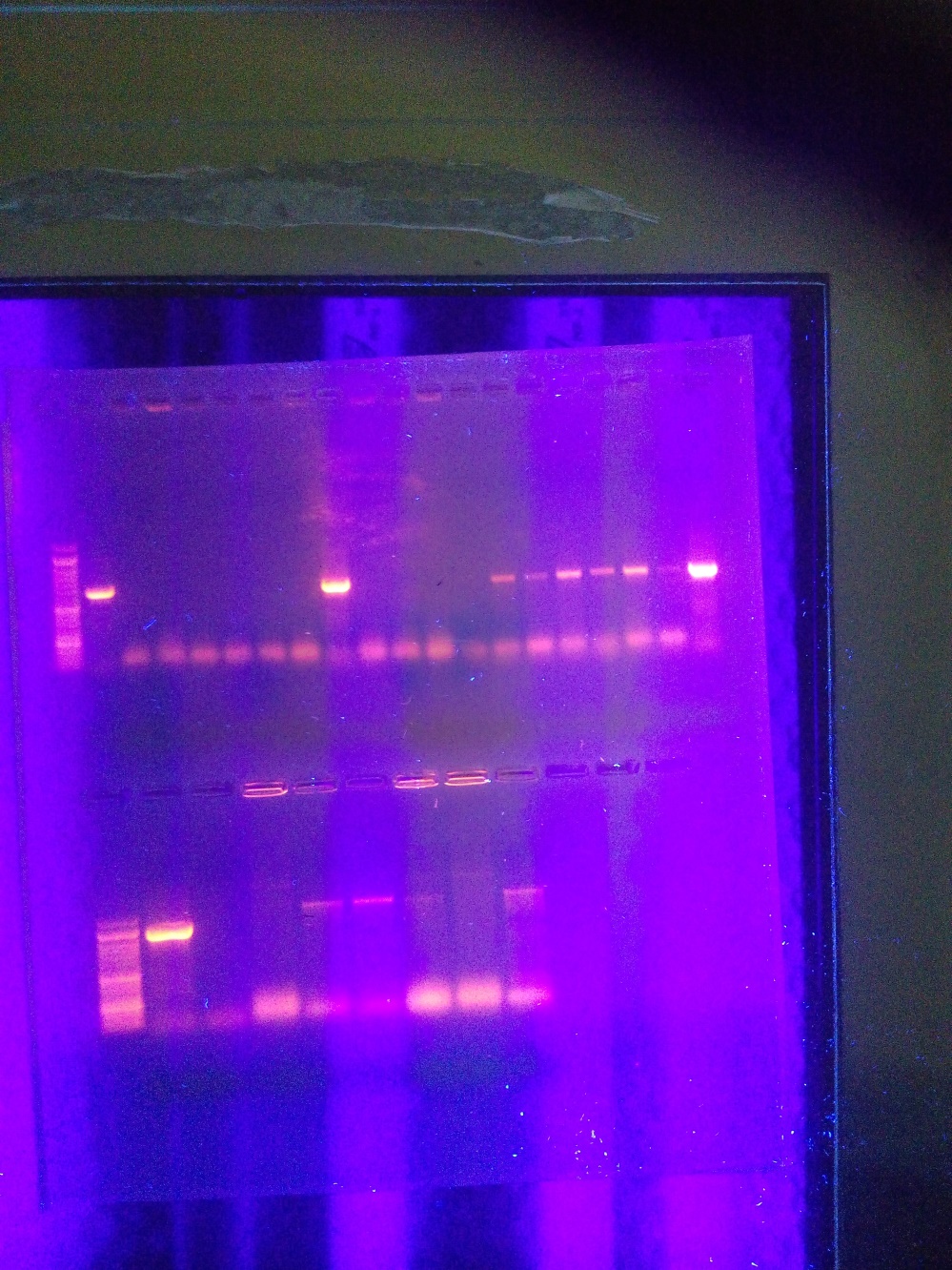
**

**Fig-3S (a)**

**
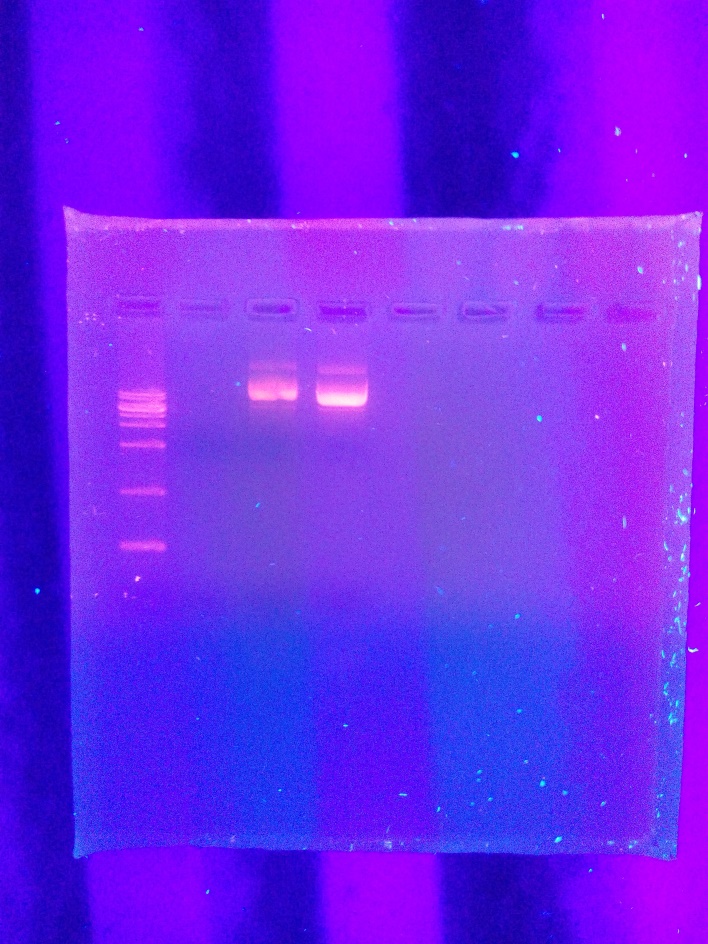
**

**Fig-3S (b)**

**
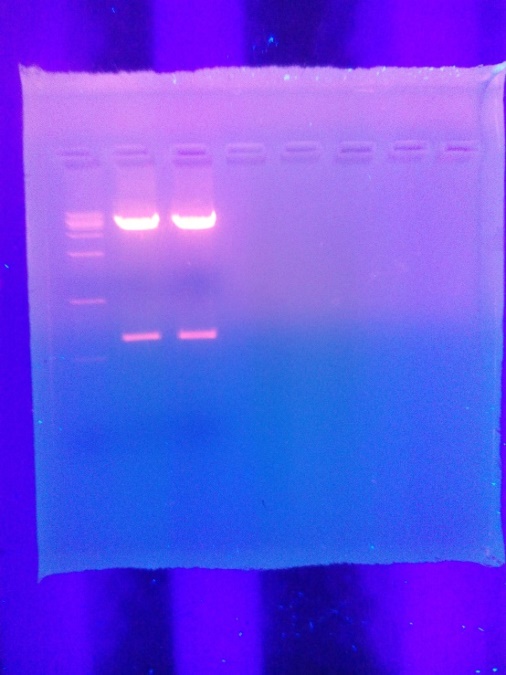
**

**Fig-3S (c)**

**
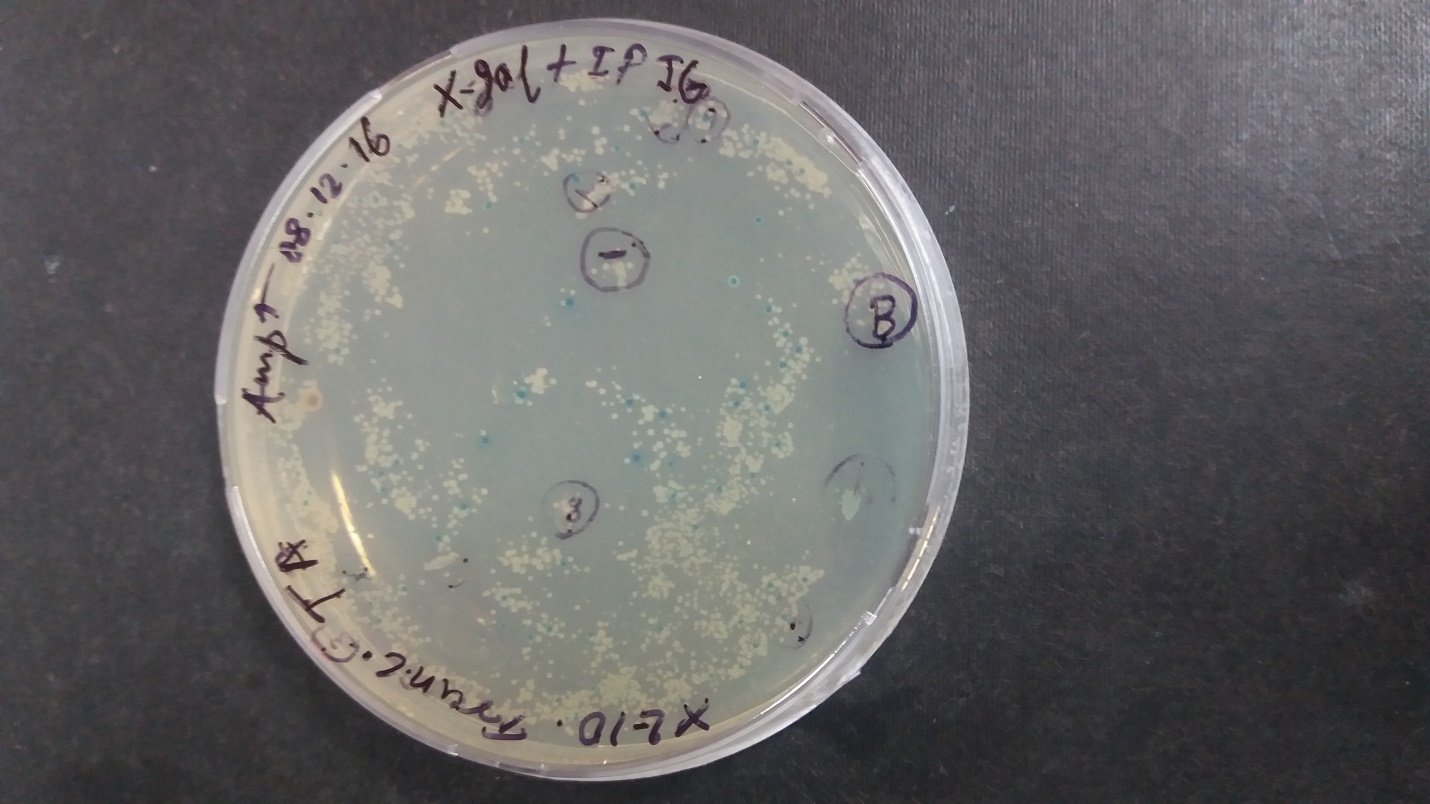
**

**Fig-4S (a)**

**
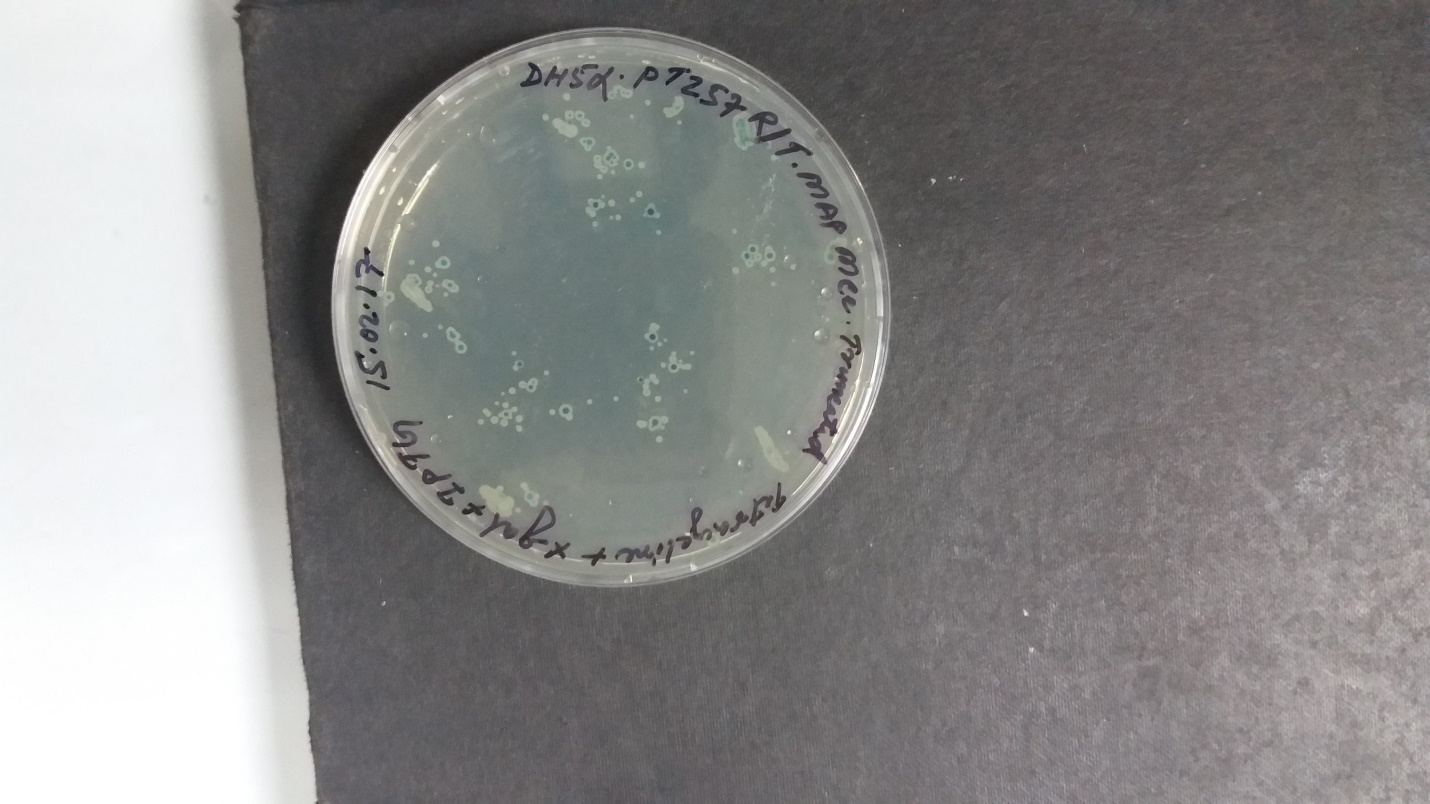
**

**Fig-4S (b)**

**
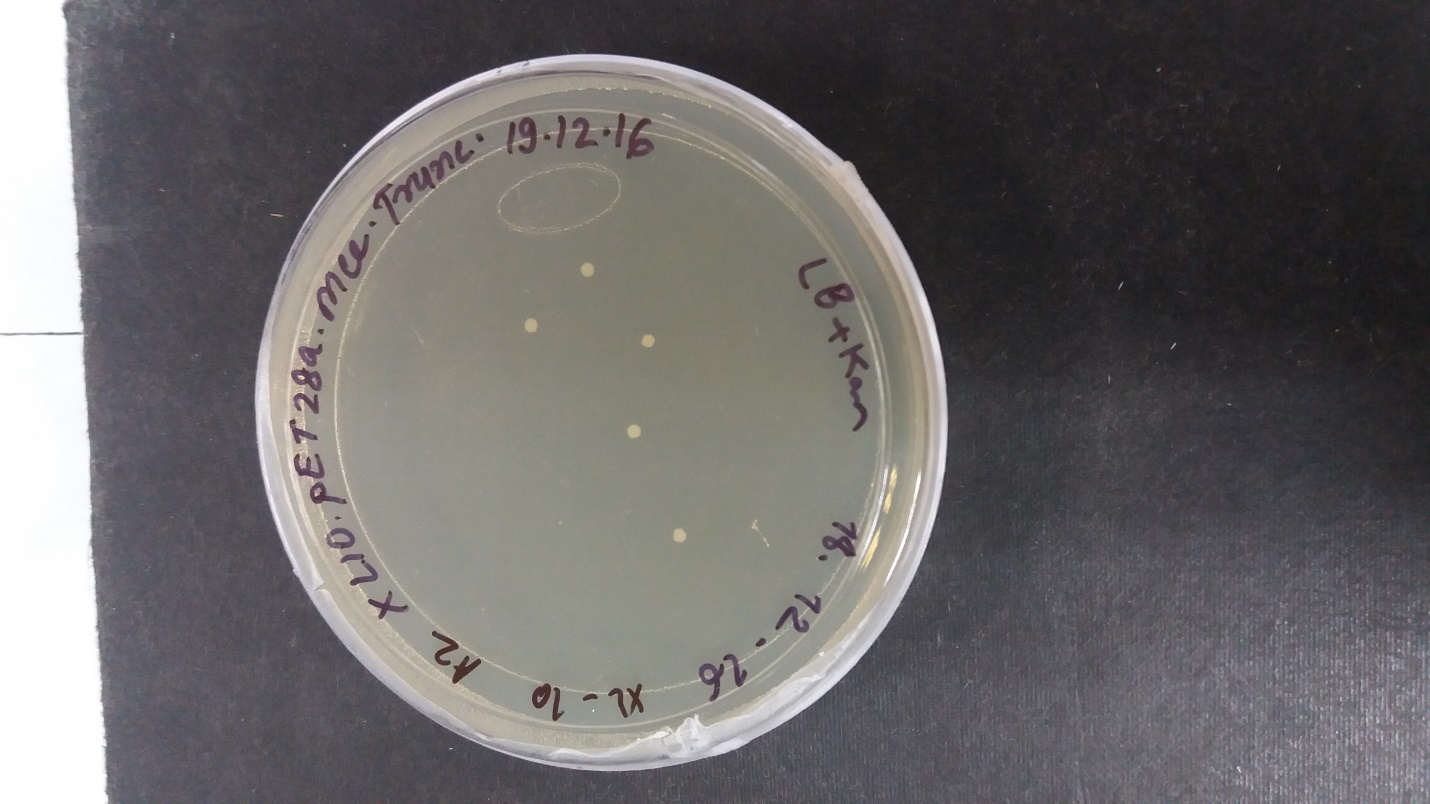
**

**Fig-4S (c)**

**
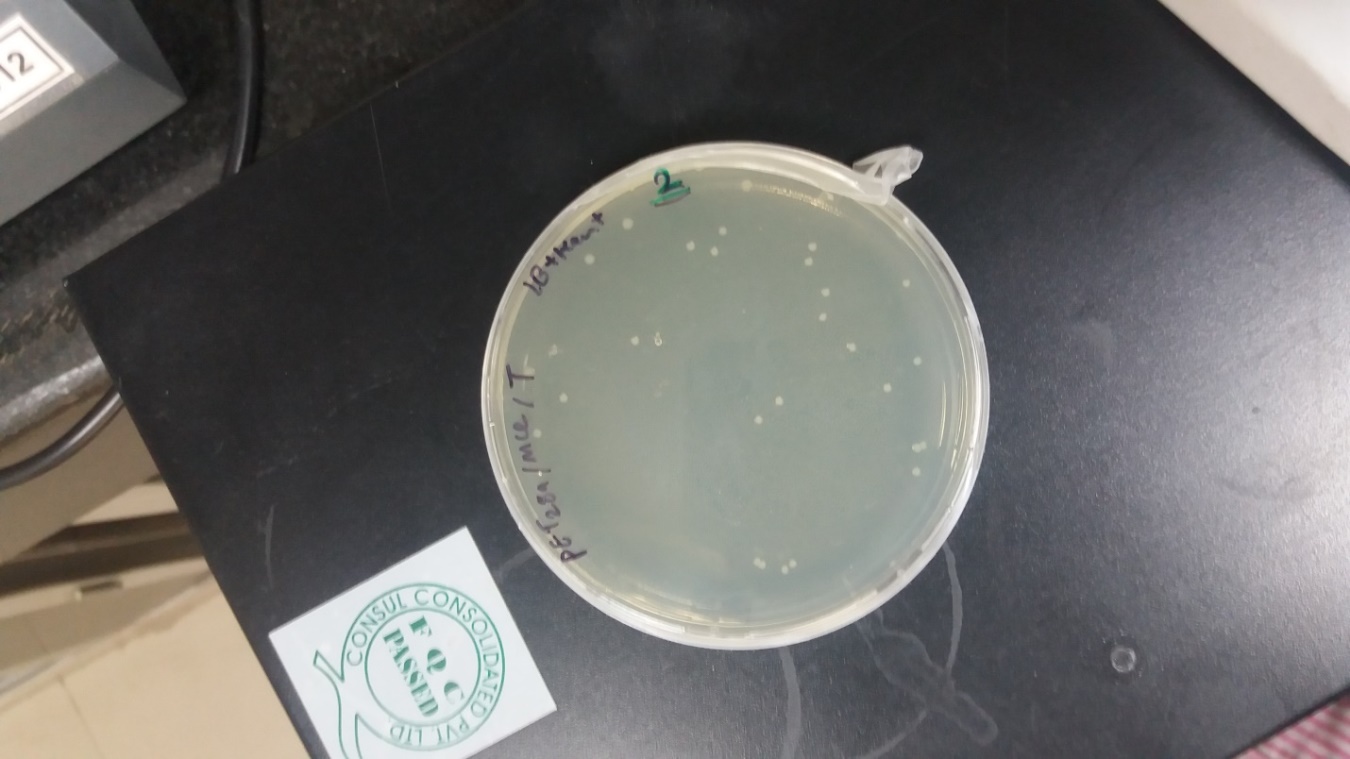
**

**Fig-4S (d)**

**
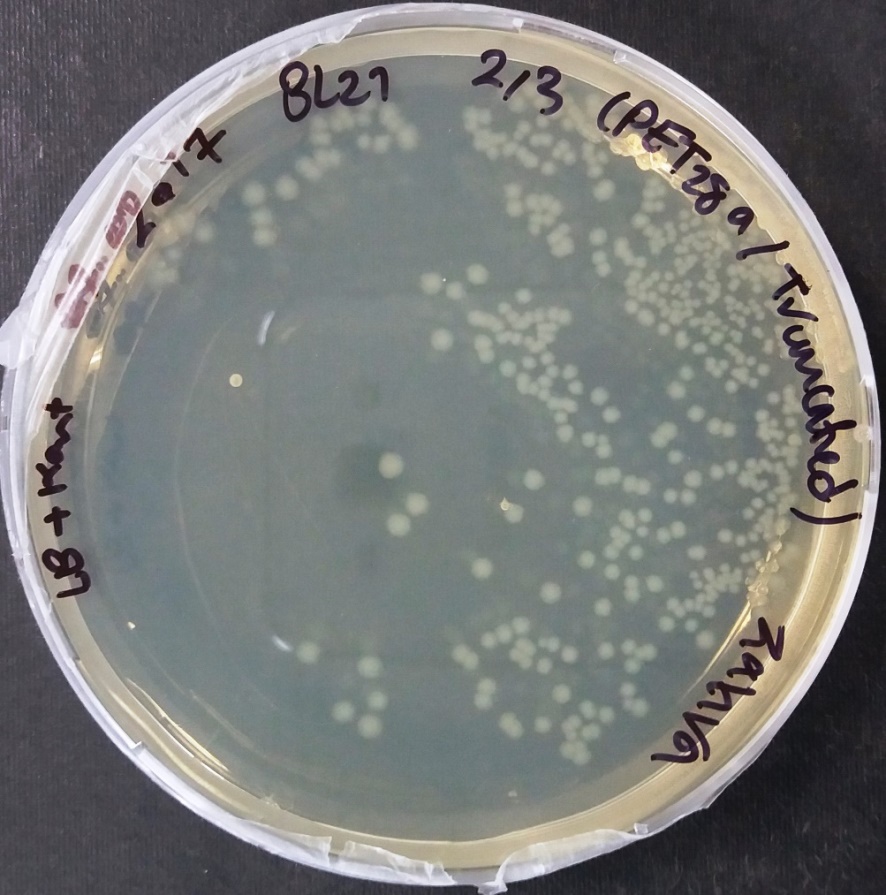
**

**Fig-4S (e)**

**
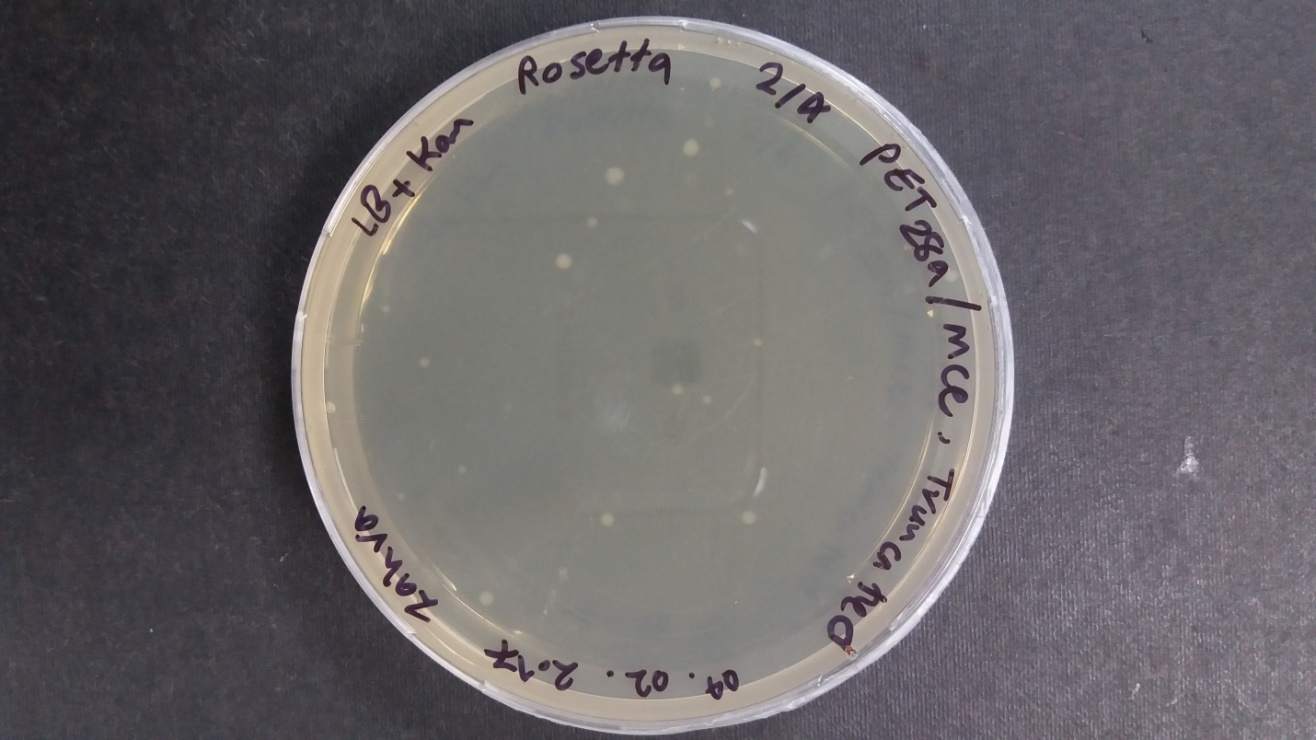
**

**Fig-4S (f)**

**
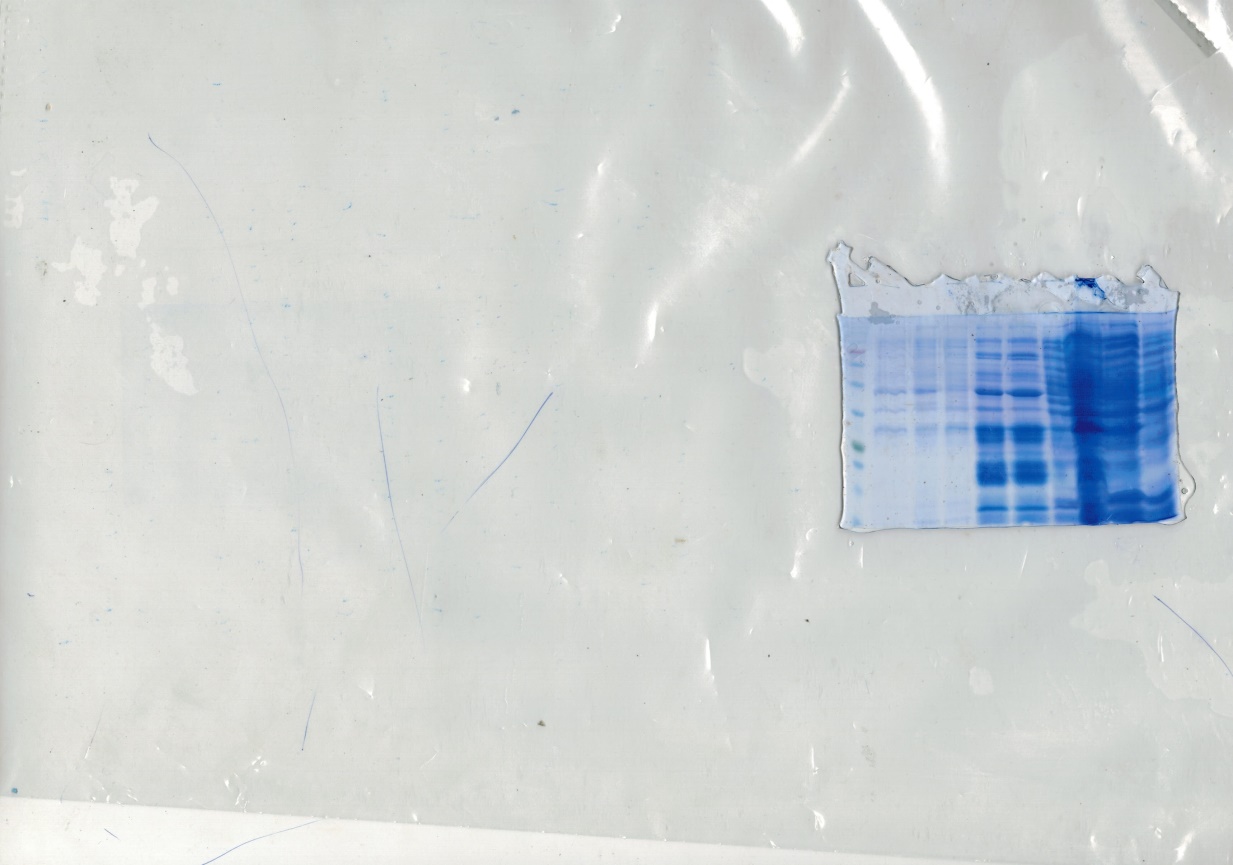
**

**Fig-5S**

**
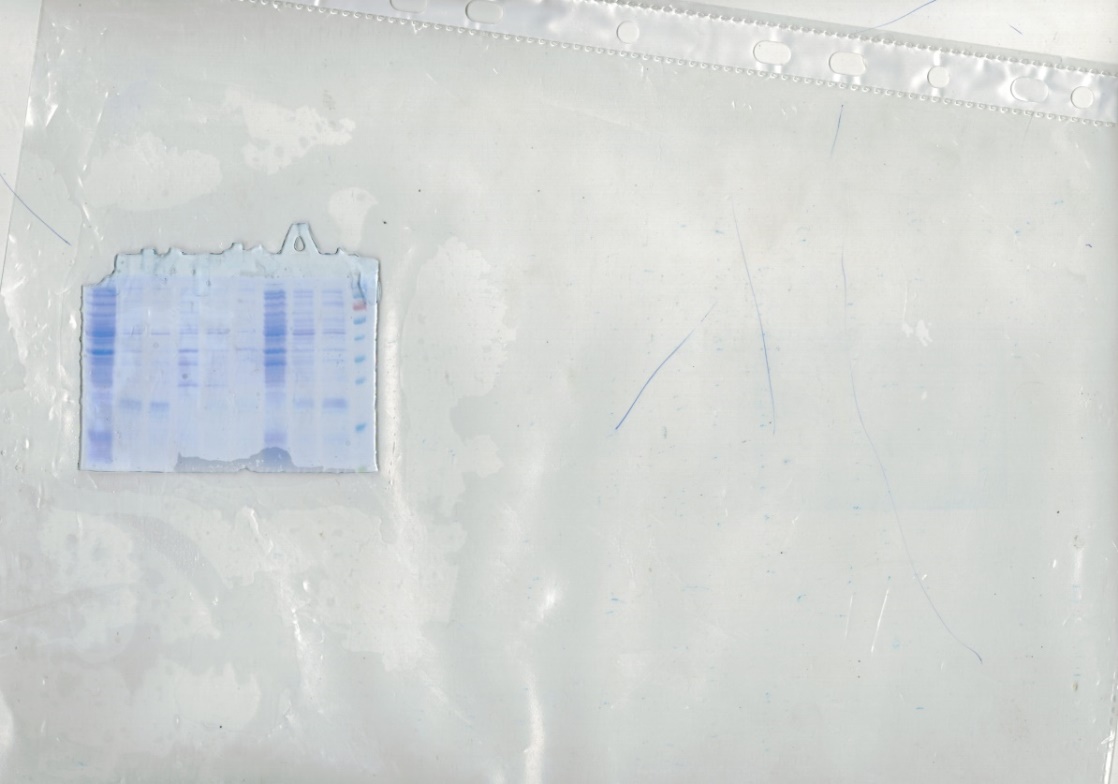
**

**Fig-6S**
